# Cathepsin B Nuclear Flux in a DNA-Guided “Antinuclear Missile” Cancer Therapy

**DOI:** 10.1021/acscentsci.4c00559

**Published:** 2024-07-15

**Authors:** Fei Cao, Caroline Tang, Xiaoyong Chen, Zewei Tu, Ying Jin, Olivia M. Turk, Robert N. Nishimura, Allen Ebens, Valentina Dubljevic, James A. Campbell, Jiangbing Zhou, James E. Hansen

**Affiliations:** † Department of Therapeutic Radiology, Yale School of Medicine, New Haven, Connecticut 06510, United States; ‡ Department of Neurosurgery, Yale School of Medicine, New Haven, Connecticut 06510, United States; § Division of Vascular Surgery and Endovascular Therapy, Department of Surgery, Yale School of Medicine, New Haven, Connecticut 06510, United States; ∥ Department of Research & Development, Greater Los Angeles Veterans Affairs Healthcare System, Los Angeles, California 90073, United States; ⊥ Department of Neurology, David Geffen School of Medicine at UCLA, Los Angeles, California 90095, United States; # Adanate, Palo Alto, California 94305, United States; 7 Patrys Ltd, Melbourne 3205, Australia; 8 Yale Cancer Center, New Haven, Connecticut 06510, United States

## Abstract

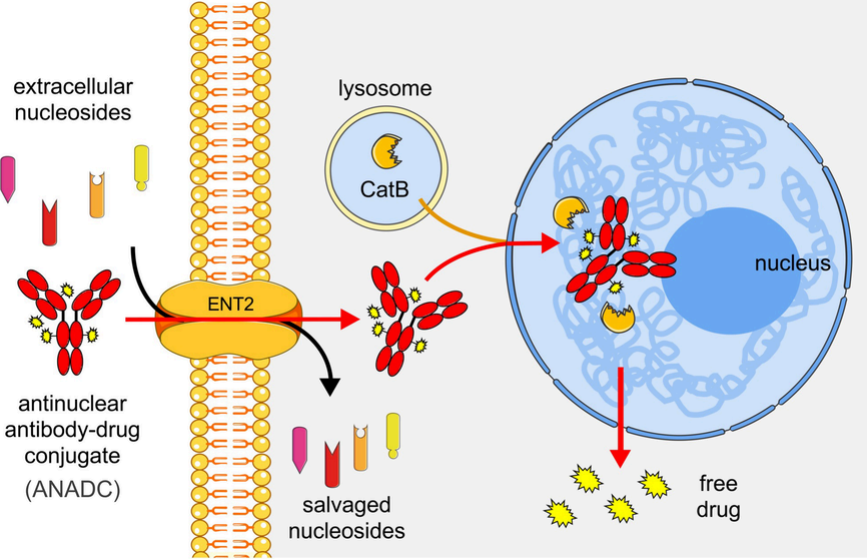

Some antinuclear antibodies (ANAs) bind extracellular nucleic acids released into tumor environments and are pulled into the nuclei of live cancer cells through nucleoside salvage pathways, independent of tumor-specific surface antigens. Here we show that ANA nuclear penetration induces nuclear flux by the lysosomal protease cathepsin B and leverage this mechanism to design an antinuclear antibody–drug conjugate (ANADC) with cathepsin B-labile drug linker. The ANADC targets nucleic acid exhaust from necrotic tumors and crosses membrane barriers through nucleoside salvage as a DNA-seeking and tumor agnostic “antinuclear missile” cancer therapy.

## Abstract 2

Some antinuclear antibodies (ANAs) in systemic lupus erythematosus bind extracellular nucleic acids and are pulled into the nuclei of nearby live cells through nucleoside salvage pathways. We now find that ANA nuclear penetration induces nuclear flux by the lysosomal protease cathepsin B. This discovery facilitates design of a tumor agnostic antinuclear antibody−drug conjugate (ANADC) that targets nucleic acid exhaust from necrotic tumors as a DNA-seeking “antinuclear missile” cancer therapy.

## Introduction

The use of antibodies as immune checkpoint inhibitors or drug delivery agents has transformed cancer care. Live cell membranes exclude most antibodies from the intracellular compartment, and mechanisms of action by most antibodies in cancer therapy share a theme of binding extracellular targets such as cell surface antigens or circulating growth factors.^1−3^ Antibody internalization by endocytosis and subsequent degradation in lysosomes after surface antigen binding has been leveraged in the design of antibody–drug conjugates (ADCs) that deliver drugs directly to cells expressing relevant surface targets such as HER2 or CD30.^4^ ADCs also have the potential to impact malignancies with low or no target expression on cancer cell surfaces by targeting microenvironmental factors such as vasculature or tumor-associated macrophages.^5−8^ In the present work we take advantage of nucleoside salvage by live tumor cells to develop an alternative method of antibody-mediated delivery of cargo drugs to tumor cells that lack specific surface antigens in an approach that overcomes membrane barriers that limit other ADCs.

Antinuclear antibodies (ANAs) are best known as markers of autoimmunity that assist clinicians in diagnosing rheumatic diseases such as systemic lupus erythematosus (SLE).^9^ The specific roles of ANAs in autoimmunity are unclear, but it is hypothesized that immune dysregulation in SLE is promoted in part by the ability of some ANAs to penetrate live cell nuclei and impact intranuclear processes. Corollary to this, nuclear-penetrating ANAs re-engineered for therapeutic applications are advancing to clinical trials.^10−13^ In contrast to most antibodies, ANAs bind extracellular nucleic acids and nucleosomes and consequently accumulate at sites of damage such as necrotic tumors, where DNA and nucleosides are released by dying cells. Some ANAs are then taken into live tumor cells through nucleoside salvage pathways mediated by transporters such as ENT2,^14−18^ allowing them to find and penetrate tumors in the absence of tumor expression of specific surface antigens. We hypothesized that the strategic design of an antinuclear antibody–drug conjugate (ANADC) that combines the tumor agnostic targeting behavior of an ANA with the toxicity of cargo drugs associated with conventional ADCs would yield a DNA-seeking “antinuclear missile” cancer therapy that does not require expression of specific surface antigens. Mechanisms responsible for degradation of nuclear-localizing antibodies were previously unknown, and we now show that ANA nuclear penetration induces nuclear translocation by the lysosomal cathepsin B protease, therein establishing a rationale for design of a nuclear-penetrating ANADC based on a cathepsin B-labile drug linker strategy. The prototype ANADC generated here penetrates and exhibits tumor agnostic effects on multiple cancer cell lines and tumors, including orthotopic intracranial glioma that is sequestered behind the blood–brain barrier (BBB).

## Results

### DX3 Is a Nuclear-Penetrating ANA

**Figure 1:**
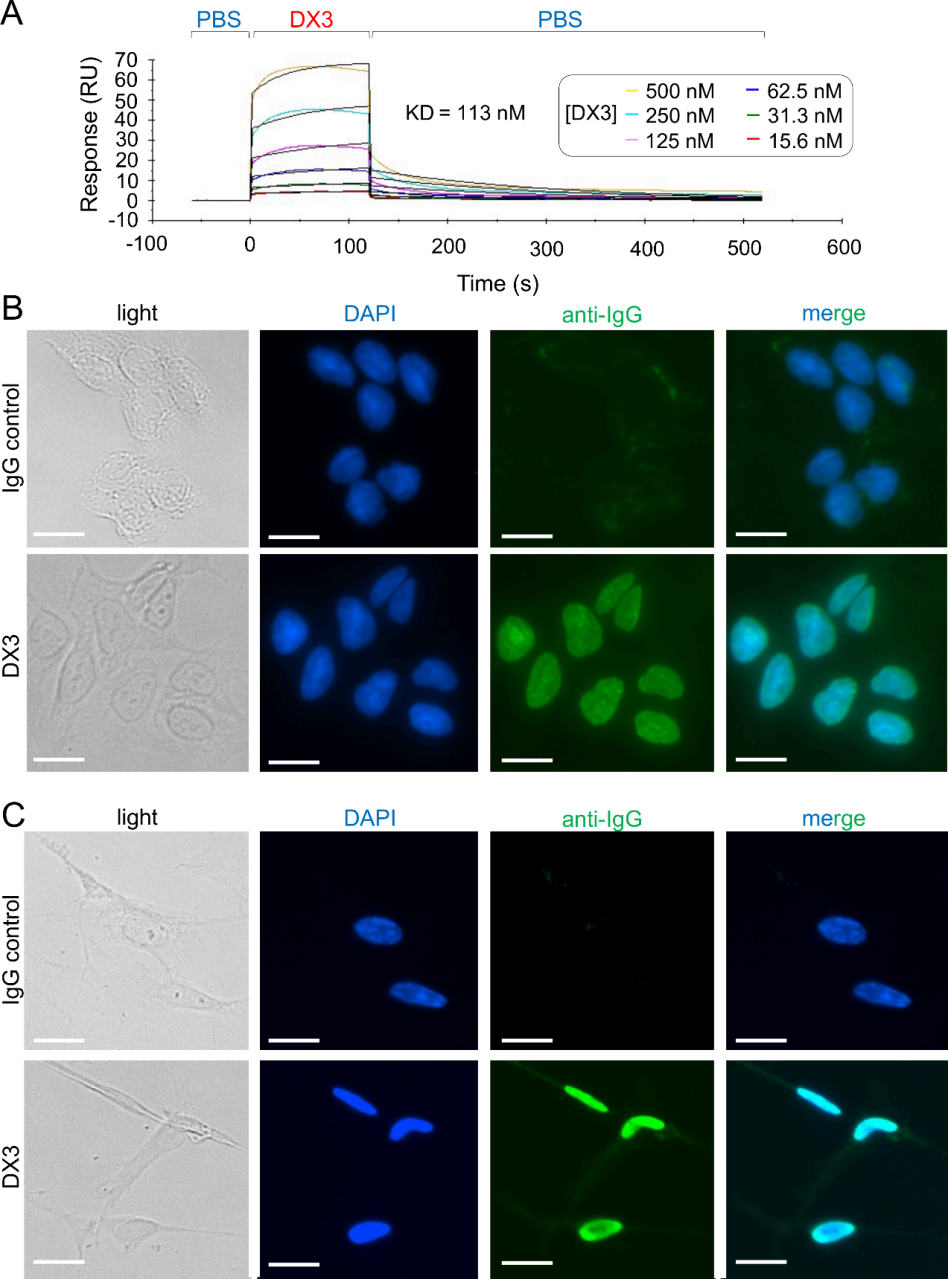
DX3 is a DNA-binding and nuclear-penetrating ANA. (A) DX3 binds single-stranded DNA. SPR binding profiles of DX3 (titrated from 500 nM) to 30-mer DNA oligonucleotide show a KD of 113 nM. (B, C) DX3 penetrates live cell nuclei. Single-channel and merged images of DLD1 colon cancer cells (B) and primary NHAs (C) treated with 4 μM IgG control or DX3 immunostained to detect antibody uptake (anti-IgG, green) with DAPI nuclear counterstain (blue) are shown. DX3 penetrated live cell nuclei, while IgG control showed minimal to no uptake into cells. Bars: 20 μm.

**Figure 2:**
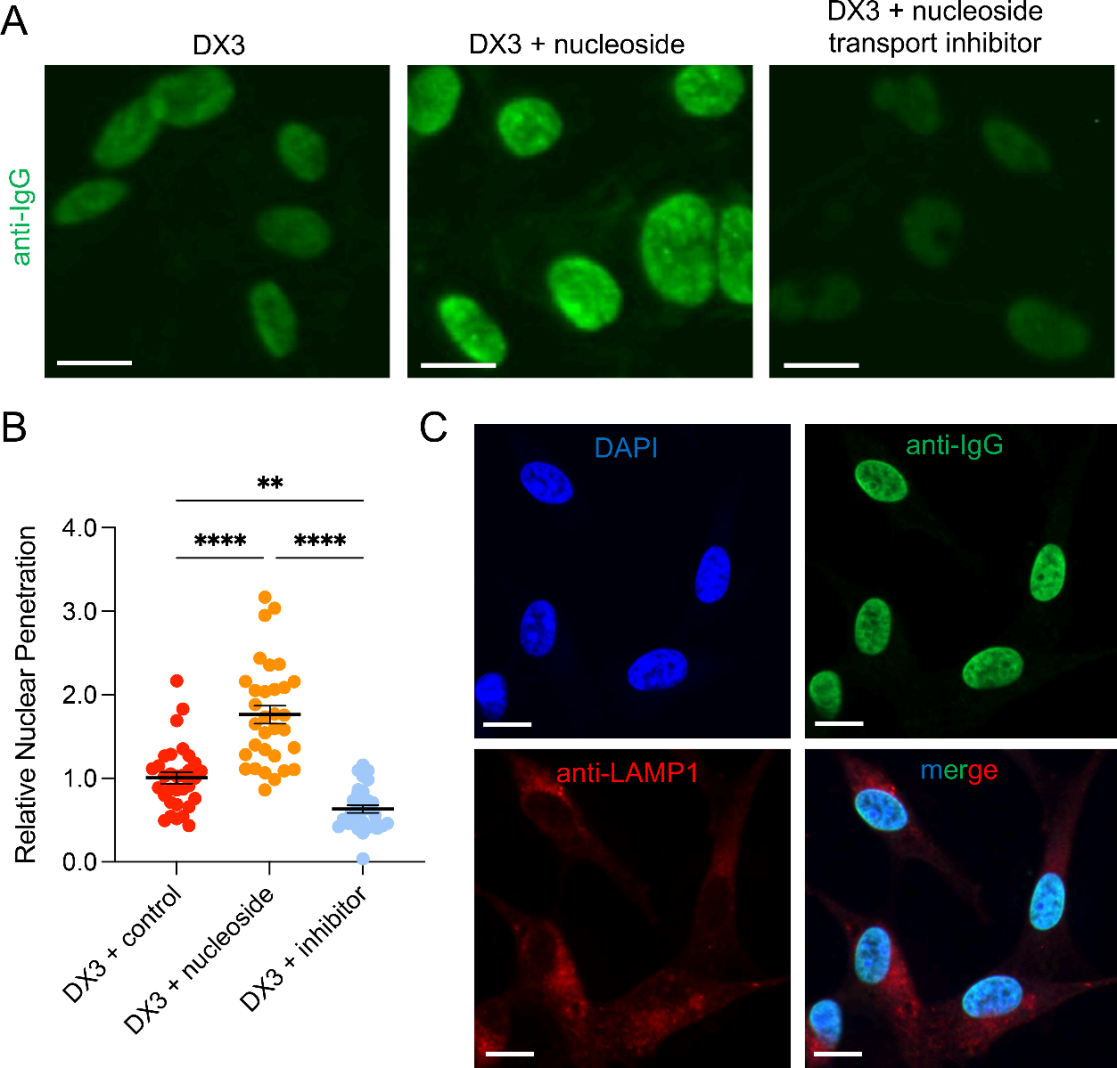
Nucleoside salvage facilitates DX3 nuclear penetration. (A) Representative images of DX3 (4 μM, 5 min treatment) uptake into U87 glioma cell nuclei in the presence of control buffer, 100 μM nucleoside (adenosine, 30 min pre-incubation), or 25 μM nucleoside transport inhibitor (dipyridamole, 30 min pre-incubation) visualized by anti-IgG immunofluorescence are shown. (B) DX3 uptake was enhanced by addition of nucleoside and suppressed by nucleoside transport inhibition, as shown by quantification of fluorescence signals by ImageJ. *N* > 30 cells per condition. **P < 0.01, ****P < 0.0001, Tukey’s multiple comparisons test. (C) Representative immunofluorescence confocal microscopy images of DX3-treated U87 glioma cells show DX3 localizes exclusively into nuclei and avoids lysosomes. DX3 was detected by anti-IgG (green), lysosomes by anti-LAMP1 (red), and nuclei by DAPI counterstain (blue). Bars: 20 μm.

The autoantibody 3E10 isolated from an MRL/lpr lupus mouse is an IgG2a kappa ANA that binds DNA, localizes to tumors, crosses cell membranes through the nucleoside transporter ENT2, and penetrates the nucleus.^10−16,18^ In the present work humanized 3E10 complementarity-determining regions (CDRs) were grafted onto a human IgG1 framework bearing the Asn297Asp (N297D) mutation that reduces FcγR interactions. The activity of the resulting antibody, hereafter referred to as Deoxymab-3 (DX3), was characterized. DX3 bound a test 30-mer single-stranded DNA oligonucleotide ligand with dissociation constant (KD) 113 nM determined by surface plasmon resonance (SPR) (Figure 1A) and penetrated the nuclei of live DLD1 colon cancer, primary normal human astrocytes (NHAs), and U87 glioma cells. A nonbinding isotype control IgG (IgG control) showed minimal to no uptake into cells (Figure 1B,C, Figure 2A).

### DX3 Promotes Nuclear Accumulation of Cathepsin B (CatB)

Mechanisms responsible for catabolism of ANAs that penetrate nuclei and avoid lysosomes are unknown. The 3E10 autoantibody from which DX3 was derived uses a nucleoside salvage-based mechanism of cellular penetration, as previously shown in gene knockout and rescue experiments and using drug inhibitors of nucleoside salvage transporters.^16,18^ The association between DX3 uptake, nucleoside transport, and lysosomal avoidance was examined. Promotion and inhibition of nucleoside transport by addition of the nucleoside adenosine or the nucleoside transport inhibitor dipyridamole enhanced and suppressed DX3 uptake, respectively (Figure 2A,B). Confocal fluorescence microscopy of DX3-treated cells immunostained for IgG and the lysosomal marker LAMP1 demonstrated DX3 nuclear penetration with lysosomal avoidance (Figure 2C). Many ADCs rely on cleavage of ADC linkers by cathepsins within lysosomes, leading to the release of payloads. Here we examined potential mechanisms of DX3 catabolism inside nuclei to explore the feasibility of using DX3 as the foundation for design of an ANADC.

**Figure 3:**
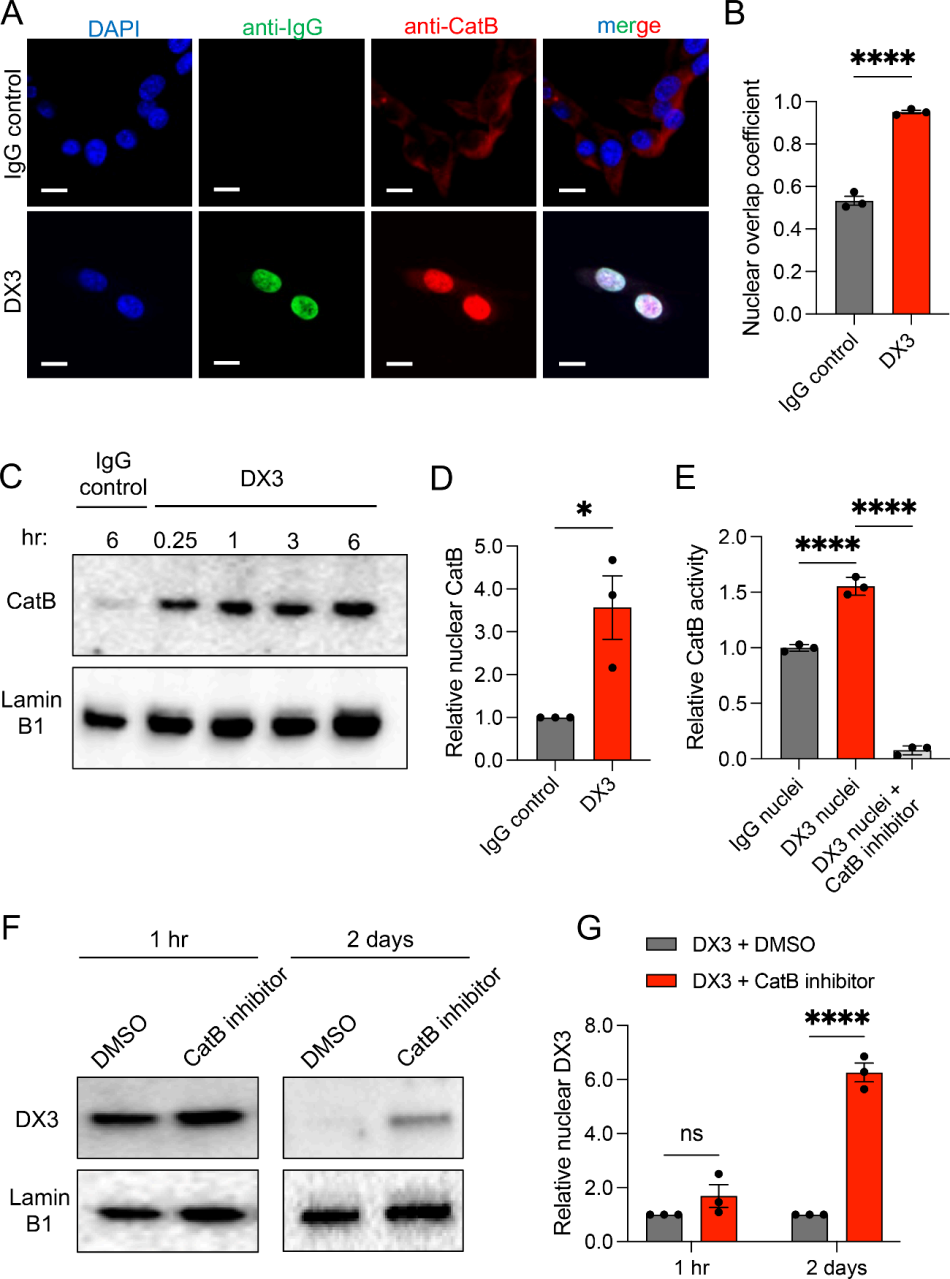
DX3 induces CatB nuclear translocation. (A) U87 glioma cells treated for 15 min with 4 μM IgG control or DX3 were immunostained for CatB (red), IgG (green), with blue DAPI nuclear counterstain. Representative confocal immunofluorescence microscopy images are shown, including single-channel and merged images. IgG control did not penetrate cells, but DX3 was taken into nuclei. CatB signal localized in the cytoplasm of cells treated with IgG control and in the nucleus of DX3-treated cells. Bars: 10 μm. Images of cells treated with control buffer and immunostained for additional cathepsins are in Figure S1. (B) Nuclear overlap coefficients for CatB in U87 glioma cells treated with control buffer, 4 μM IgG control, or 4 μM DX3 were determined by ImageJ Colocalization Finder. DX3 induced a significant increase in CatB nuclear overlap compared to control buffer or IgG control. ****P < 0.0001, two-tailed Student’s *t* test, *n* = 3. (C, D) Nuclear contents isolated from U87 glioma cells treated for 0–6 h with 4 μM IgG control or DX3 were analyzed by western blot probed for CatB, with Lamin B1 (∼66 kDa) for loading control. DX3 caused a significant increase in nuclear content of the active form of CatB (∼29 kDa). Representative western blot on nuclear extracts is shown in (C), and ImageJ quantification of CatB nuclear content relative to content in cells treated with IgG control at 6 h is shown in (D) respectively. *P < 0.05, two-tailed Student’s *t* test, *n* = 3. (E) CatB activity is increased in nuclear extracts from DX3-treated cells. Cleavage of a CatB fluorogenic substrate by nuclear extracts from U87 cells treated with IgG control (IgG nuclei), DX3 (DX3 nuclei), or DX3 + CA-074Me CatB inhibitor (DX3 nuclei + inhibitor) was measured by fluorescence plate reader. ****P < 0.0001, Tukey’s multiple comparisons test, *n* = 3. (F, G) Nuclear extracts of U87 cells exposed to DX3 in the presence of DMSO control or the CatB inhibitor CA-074Me were probed by western blot for DX3 content 1 h or 2 days after application of DX3. Representative western blots are shown in (E) and ImageJ quantification of relative DX3 content is shown in (D), respectively. ****P < 0.0001, two-tailed Student’s *t* test, *n* = 3.

Some lysosomal cathepsin proteases are released into the cytoplasm and translocate into the nucleus and are implicated in mechanisms of nucleocytoplasmic shuttling.^19−23^ Intracellular localization of cathepsins involved in nuclear import and export (CatB, CatK, CatL, and CatS)^19^ were examined in U87 glioma cells 15 min after treatment with buffer control, IgG control, or DX3 by confocal fluorescence microscopy. IgG control did not impact localization of these cathepsins, but DX3 caused a marked shift of CatB signal into the nucleus (Figure 3A, Figure S1A,B). To quantitatively evaluate this effect, nuclear overlap coefficients were determined for each of the cathepsins in IgG control and DX3-treated cells using ImageJ Colocalization Finder. CatB nuclear overlap coefficient was increased to 0.95 ± 0.01 in DX3-treated cells compared to 0.53 ± 0.02 in cells treated with IgG control (*P* < 0.0001), while coefficients were unchanged by DX3 for CatK and CatL and only minimally increased for CatS (Figure 3B, Figure S1C). Western blots with ImageJ quantification of bands on nuclear and cytoplasmic extracts from IgG control or DX3-treated U87 cells confirmed DX3 promotes nuclear accumulation of the active isoform of CatB (molecular weight ∼29 kDa) with a corresponding decrease in cytoplasmic content of active CatB (Figure 3C,D, Figure S2). Consistent with this, CatB enzymatic activity was increased in nuclear extracts from DX3 compared to IgG control-treated U87 cells, and this activity was suppressed by pretreatment of cells with the CatB inhibitor CA-074Me^24^ (Figure 3E). The impact of DX3 on CatB nuclear flux was conserved over a panel of cancer cell lines including glioma, breast, colon, and lung cancer cells and primary cells (Figures S3, S4).

The effect of CatB inhibition on DX3 stability in the nucleus was probed to determine if CatB is involved in ANA nuclear catabolism. U87 cells were treated with DX3 in the presence of DMSO control or the CatB inhibitor CA-074Me. One hour after DX3 application, media were removed and replaced with fresh media lacking DX3 but containing DMSO or CA-074Me. Intranuclear DX3 content was then measured by western blot on nuclear extracts taken at time points of 1 h or 2 days after initial exposure to DX3. CatB inhibition significantly delayed the intranuclear degradation of DX3 (Figure 3F,G). Taken together, these data demonstrate that active CatB rapidly accumulates in the nucleus of DX3-treated cells and is associated with degradation of DX3, indicating a CatB role in intranuclear catabolism of ANAs.

### Leveraging CatB Nuclear Translocation to Design an Antinuclear Antibody–Drug Conjugate

**Figure 4:**
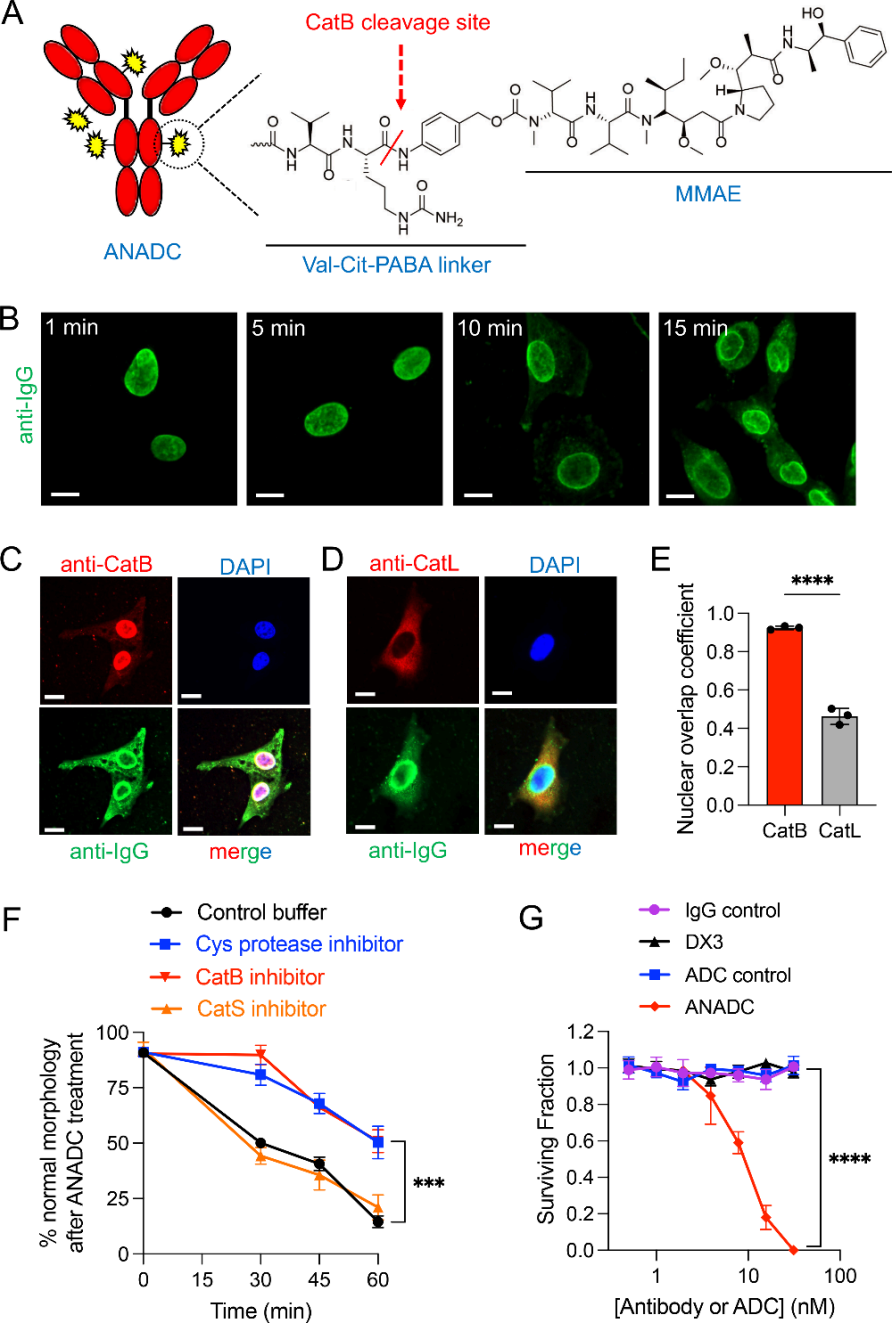
ANADC causes CatB nuclear translocation and CatB-linked cytotoxicity. (A) Schematic illustrating a CatB-labile dipeptide VC-based linker joining MMAE to DX3 to form ANADC. (B) Confocal immunofluorescence microscopy images of ANADC-treated U87 glioma cells stained for IgG (green) show ANADC localizes exclusively in the nucleus at early time points, followed by the appearance of some cytoplasmic signal at later time points. Bars: 10 μm. (C–E) ANADC induces CatB nuclear translocation. Immunofluorescence confocal microscopy images of U87 glioma cells treated with ANADC for 30 min stained for IgG (green) and CatB or CatL (red) with DAPI nuclear counterstain (blue) show CatB (C) but not CatL (D) colocalizing with DX3 and DAPI in the nucleus. Bars: 10 μm. Nuclear overlap coefficients quantified by ImageJ Colocalization Finder confirmed ANADC induced nuclear translocation by CatB but not CatL. ****P < 0.0001, two-tailed Student’s *t* test, *n* = 3 (E). (F) The association of ANADC cytotoxicity with CatB function was tested. U87 glioma cells preincubated with control buffer (DMSO) or 10 μM protease inhibitors (E64 Cys protease inhibitor, CA-074Me CatB inhibitor, or LY3000328 CatS inhibitor) were treated with control media or 4 μM ANADC ± inhibitors. Cells were monitored by bright-field microscopy with quantification of morphologic changes by ImageJ. The Cys protease inhibitor (which inhibits CatB and other Cys proteases) and the CatB inhibitor provided protection to the cells compared to control buffer or the CatS inhibitor, consistent with CatB-dependent cytotoxicity. ***P < 0.001, Tukey’s multiple comparisons test, *n* = 3. (G) ANADC is toxic to U87 glioma cells. Cells were treated with titrated doses of IgG control, ADC control, DX3, and ANADC, and surviving fractions determined by colony formation assay. ****P < 0.0001, Tukey’s multiple comparisons test, *n* = 3.

Discovery of DX3 induction of CatB nuclear translocation offered a rationale for design of a first-in-class ANADC based on leveraging the accumulation of CatB in the nucleus of cells treated with a DX3-based ANADC to facilitate release of drug from a CatB-labile drug linker. The dipeptide valine-citrulline (VC) linker, which is used in clinical stage conventional ADCs such as brentuximab vedotin,^25^ was originally designed to be specifically cleavable by CatB, although subsequent work suggests it may also be cleaved by CatB-independent processes.^26,27^ Based on its previous success in the clinic we used the VC linker strategy to develop a DX3-based ADC (hereafter referred to as ANADC) (Figure 4A).

ANADC was generated by maleimide-based linkage of DX3 to the antimitotic agent monomethyl auristatin E (MMAE) with incorporation of the CatB-labile VC dipeptide and self-immolative PABA spacer. MMAE was selected based on its previous successful use with this linker in conventional ADCs.^25^ A nonbinding isotype control IgG linked to MMAE was prepared using the same protocol and is hereafter called ADC control. Purity and absence of free drug was confirmed by size exclusion high-performance liquid chromatography (SEC-HPLC) and liquid chromatography mass spectrometry (LC-MS). Both ADCs were >98% pure with undetectable free drug, and drug–antibody ratios (DARs) were closely matched between ANADC and ADC control at 4.5–4.8 and 4.7–4.9 as determined by LC-MS and hydrophobic interaction chromatography (HIC), respectively (Figure S5).

ANADC activity in cell culture was characterized. U87 glioma cells treated with IgG control, ADC control, unconjugated DX3, or ANADC for 60 min were washed, fixed, and immunostained to detect antibody uptake. IgG control and ADC control showed minimal uptake into cells. Unconjugated DX3 localized into nuclei, while ANADC signal was detected in both nuclear and cytoplasmic compartments, including some punctate foci of staining in the cytoplasm. Bright-field examination of cell morphologies showed loss of normal contours in ANADC-treated cells compared to cells treated with IgG control, ADC control, or unconjugated DX3. These findings suggested cell death caused by MMAE released from ANADC allowed some DX3 to leak out of the nucleus into the cytoplasm that was detectable by immunostaining (Figure S6A). To further probe this effect, ANADC localization in U87 cells was examined at earlier time points. At 1 and 5 min after treatment ANADC signal was exclusively in the nucleus and did not overlap with β-tubulin or lysosomes, indicating that linkage to MMAE did not alter the initial intracellular trafficking of DX3 into the nucleus. Progressive leakage of antibody signal into the cytoplasm was observed at later time points (Figures 4B, S6B, S7, S8) that correlate with cells beginning to die based on morphology changes. The ability of ANADC to induce CatB nuclear accumulation in glioma cells was confirmed by fluorescence confocal microscopy (Figure 4C–E).

We hypothesized that CatB-mediated cleavage of the VC linker in ANADC facilitates release of the cytotoxic MMAE cargo drug. To test this, the protective effect of a CatB inhibitor against the cytotoxicity of subsequent ANADC treatment was examined. U87 cells pretreated with control buffer or E64 Cys protease inhibitor, CA-074Me CatB inhibitor, or LY3000328 CatS inhibitor were incubated in control media or media containing ANADC ± the inhibitors listed above. Toxicity was followed by bright-field microscopy and quantification of morphologic changes by ImageJ. ANADC rapidly induced loss of cell morphologies in the presence of control buffer or the CatS inhibitor. In contrast, the broad-spectrum Cys protease inhibitor (which has an inhibitory effect on CatB and other Cys proteases) and the CatB inhibitor significantly suppressed these morphologic changes, consistent with CatB-dependent cytotoxicity (Figures 4F, S9). The effects of IgG control, ADC control, unconjugated DX3, and ANADC on the survival of U87 glioma cells were then evaluated in colony formation assays. Cells tolerated IgG control, ADC control, and unconjugated DX3 over a dose range of 0.5 to 31.25 nM, but ANADC was highly toxic to the cells at concentrations above 7 nM (Figure 4G). ANADC penetration and cytotoxicity were consistent in additional cancer cells including triple-negative MDA-MB-231 and ER+ MCF7 breast, DLD1 colon, and A549 lung cancer cells (Figure S10).

### ANADC Suppresses Subcutaneous Xenograft Tumors

**Figure 5:**
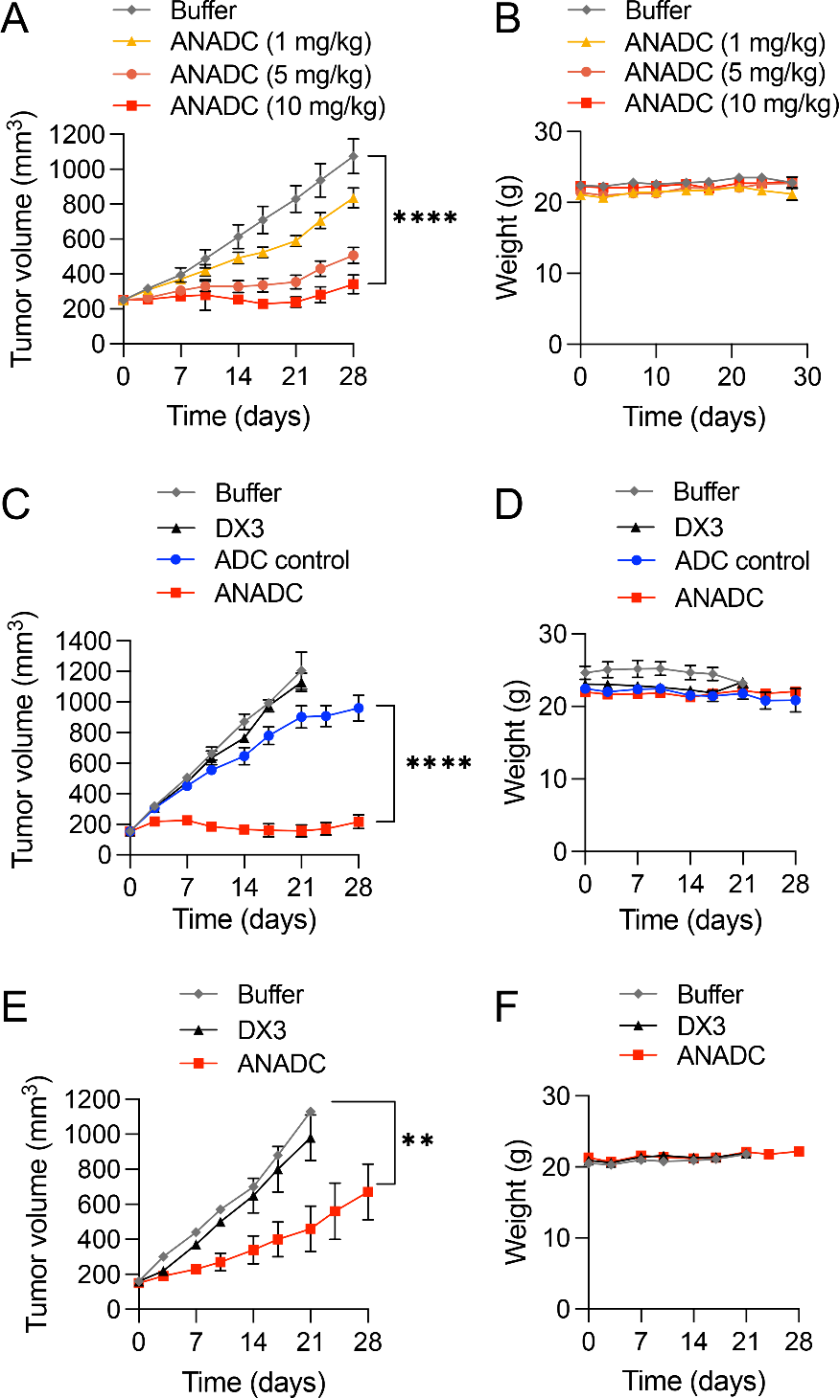
ANADC suppresses tumor growth in multiple subcutaneous xenograft models. (A, B) ANADC suppresses triple-negative breast cancer tumor growth in a dose-dependent manner. Tumor volumes in mice bearing MDA-MB-231 subcutaneous xenograft flank tumors treated with four weekly IP injections of control buffer or ANADC at 1, 5, or 10 mg/kg (*n* = 8 per group) once a week for 4 weeks (treatments on days 0, 7, 14, and 21 in the figure) are shown in (A) and body mass measurements over the study in (B). ANADC showed dose-dependent suppression of tumor growth (****P < 0.0001, Tukey’s multiple comparison test) and was well tolerated at all doses. Based on this, a dose of 10 mg/kg was selected for subsequent studies. (C, D) ANADC suppresses ER+ breast cancer tumor growth. Tumor volumes in mice bearing MCF7 subcutaneous xenograft flank tumors treated with four weekly IP injections of control buffer, nonbinding ADC control (10 mg/kg), unconjugated DX3 (10 mg/kg), or ANADC (10 mg/kg) (*n* = 6 per group) once a week for 4 weeks (treatments on days 0, 7, 14, and 21 in the figure) are shown in (C) and body mass measurements over the study in (D). ANADC showed the greatest inhibition of tumors (****P < 0.0001, Tukey’s multiple comparison test) and was well tolerated. (E, F) ANADC suppresses colon cancer tumor growth. Tumor volumes in mice bearing HT29 subcutaneous xenograft flank tumors treated with four weekly IP injections of control buffer, unconjugated DX3 (10 mg/kg), or ANADC (10 mg/kg) (*n* = 6 per group) once a week for 4 weeks (treatments on days 0, 7, 14, and 21 in the figure) are shown in (E) and body mass measurements over the study in (F). ANADC significantly suppressed tumor growth (**P < 0.01, Tukey’s multiple comparison test) and was well tolerated.

ANADC efficacy was tested in multiple subcutaneous xenograft tumor models. In a dose-finding study, mice bearing MDA-MB-231 triple-negative breast cancer subcutaneous tumors were randomized to treatment with four weekly intraperitoneal (IP) injections of control buffer or ANADC at 1, 5, or 10 mg/kg (*n* = 8 mice per group). Study end points were tumor volume or changes in behavior or weights. ANADC yielded a dose-dependent suppression of tumor growth compared to control buffer (****P < 0.0001, Tukey’s multiple comparison test) (Figure 5A) and was well tolerated at all doses (Figure 5B). Based on this, an ANADC dose of 10 mg/kg was selected for use in subsequent studies. ANADC efficacy was compared against control buffer, unconjugated DX3, and nonbinding ADC control in a second breast cancer model (ER+ MCF7 breast cancer). Mice with subcutaneous MCF7 tumors were randomized to treatment with four weekly IP injections of control buffer, unconjugated DX3 (10 mg/kg), ADC control (10 mg/kg), or ANADC (10 mg/kg) (*n* = 6 mice per group). As shown in Figure 5C, tumor suppression by ANADC was significantly greater than all controls (****P < 0.0001, Tukey’s multiple comparison test), and ANADC was well tolerated (Figure 5D). Next, ANADC efficacy against tumors from colon cancer was tested in the HT29 model, with mice randomized to four weekly IP injections of control buffer, unconjugated DX3 (10 mg/kg), or ANADC (10 mg/kg) (*n* = 6/group). Consistent with the breast cancer studies, ANADC significantly suppressed tumor growth and was well tolerated, with no significant differences in body mass observed throughout treatment (Figure 5E,F).

### DX3 Localizes to Brain Tumors and ANADC Prolongs Survival in an Orthotopic Glioma Model

**Figure 6:**
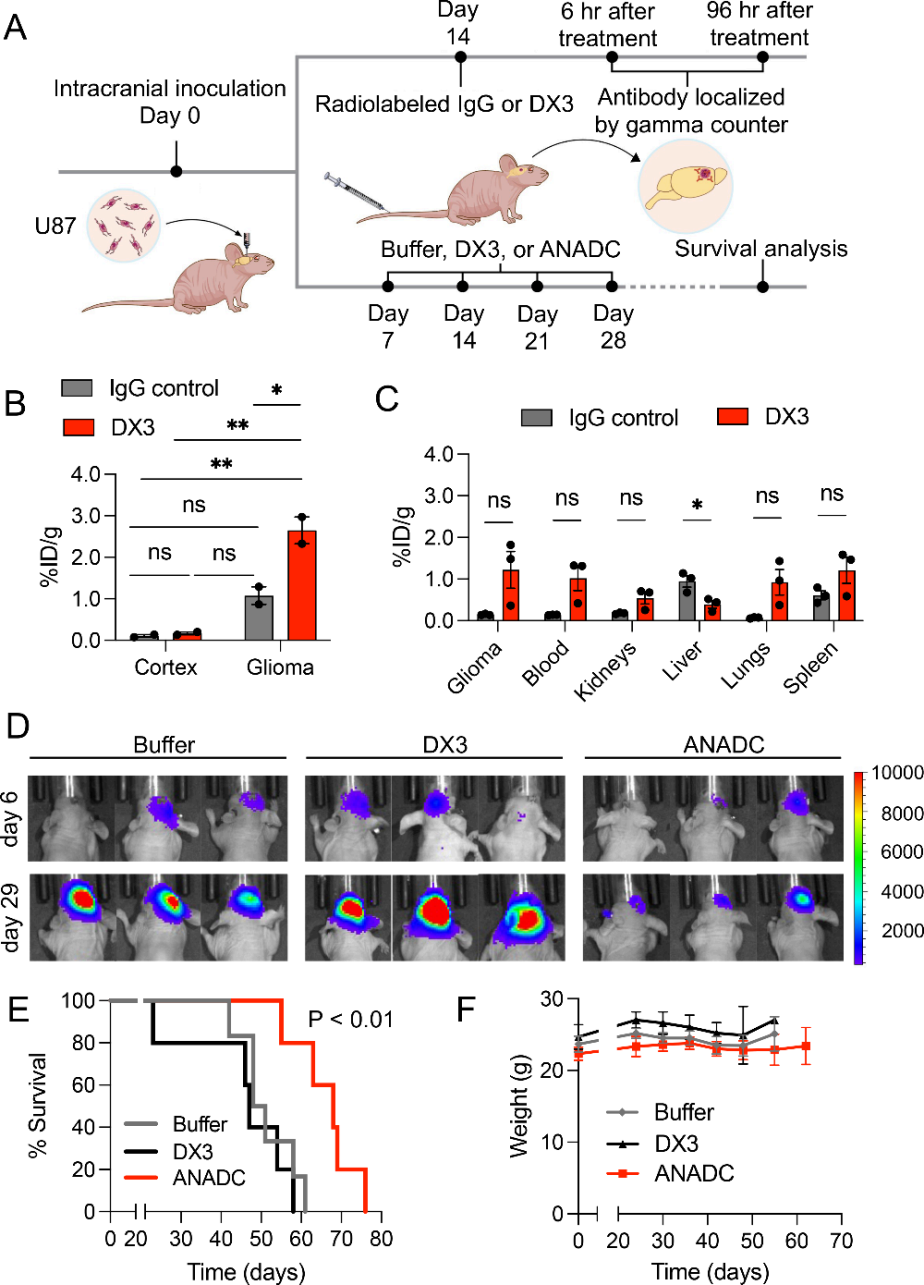
DX3 localizes to brain tumors and ANADC prolongs survival in an orthotopic glioma model. (A) Schematic showing design of parallel brain tumor localization and survival studies in an orthotopic GBM model. (B) DX3 preferentially localizes to brain tumors. Brains were taken from mice bearing intracranial U87 glioma tumors 6 h after treatment with radiolabeled IgG control or DX3. Tumor and normal brain cortex and other components were separately analyzed by gamma counter to quantify antibody uptake. Antibody uptake in glioma compared to normal brain cortex is shown. Results are expressed as %ID/g. *n* = 2. *P < 0.05, **P < 0.01, Tukey’s multiple comparisons test. (C) Systemic distribution of DX3 in mice with brain tumors. Tissues were taken from mice bearing intracranial U87 glioma tumors 96 h after treatment with radiolabeled IgG control or DX3 and analyzed by gamma counter to quantify DX3 uptake relative to IgG control. Compared to IgG control, DX3 content was decreased in livers. No other statistically significant differences were identified. Results are expressed as %ID/g. *n* = 3, *P < 0.05, two-tailed Student’s *t* test. (D–F) ANADC prolongs survival in an orthotopic glioma model. Mice bearing intracranial U87 glioma tumors were treated with four weekly tail vein injections of control buffer (*n* = 6), DX3 (10 mg/kg, *n* = 5), or ANADC (10 mg/kg, *n* = 5). Mice were followed for end points of neurologic changes or weight loss. Representative IVIS images at day 29 (1 day after completion of treatment) are shown in (D). Kaplan–Meier plots show median survivals of 49.5, 47, and 68 days associated with control buffer, DX3, and ANADC, respectively (*P* < 0.01, log-rank test) (E). All treatments were well tolerated based on stable body masses (F).

The BBB limits the direct access of most antibodies and ADCs to brain tumors. The nucleoside transporter ENT2 manages nucleoside flux at the BBB and was previously shown to facilitate BBB crossing and brain tumor localization by a 3E10 fragment.^18^ Based on this, we hypothesized that DX3 would localize to orthotopic brain tumors and that the DX3-based ANADC would deliver cargo drug to prolong survival in an orthotopic brain tumor model. The impact of brain tumor localization by DX3 and ANADC on survival in a U87 glioma model was evaluated in parallel studies (Figure 6A). Biodistribution and trafficking to brain tumors by DX3 or IgG control were evaluated in NMRI-Foxn1^nu^ mice. Mice bearing orthotopic U87 brain tumors confirmed by MRI were randomized to treatment with ^125^I-radiolabeled IgG control (*n* = 5) or DX3 (*n* = 5). Brains were taken 6 h after treatment from two mice in each group, and tumors and normal brain regions separated for analysis of antibody uptake by gamma counter. Results expressed as percentage of injected dose/gram of tissue (%ID/g) showed increased DX3 localization into brain tumor compared to IgG control (%ID/g 2.7 ± 0.3 versus 1.1 ± 0.2, *P < 0.05) but not into normal brain cortex or other regions of normal brain (Figures 6B, S11). Brains and additional normal tissues (blood, kidneys, liver, lungs, spleen) from the remaining three mice in each group were taken for gamma counting at 96 h post-treatment. No statistically significant differences between IgG control and DX3 were detected in the examined tissues except for the liver, where DX3 content was reduced (*P < 0.05, two-tailed Student’s *t* test) (Figure 6C). These data indicate DX3 bioavailability to orthotopic brain tumors without significant off-target deposition compared to IgG control.

In the survival study, athymic nude mice bearing orthotopic luciferase expressing U87 brain tumors confirmed by an *in vivo* imaging system (IVIS) were randomized to treatment with four cycles of weekly tail vein injections of control buffer (*n* = 6), DX3 (10 mg/kg) (*N* = 5), or ANADC (10 mg/kg) (*n* = 5) (Figure 6A). Mice were monitored and euthanized for weight loss or neurologic changes. IVIS images taken day 29 (1 day after the last treatments) showed an apparent suppression of tumor growth associated with ANADC (Figure 6D). ANADC significantly prolonged survival compared to buffer or unconjugated DX3, with median survivals of 68, 49.5, and 47 days, respectively (*P* < 0.01, log-rank test) (Figure 6E). All treatments were well tolerated based on stability of behaviors and body mass (Figure 6F). These data demonstrate the bioavailability of DX3 to and efficacy of ANADC against orthotopic brain tumors.

## Discussion

The ANADC developed here targets the nucleic acid exhaust released by necrotic tumor cells^15,28,29^ and exploits mechanisms of nucleoside salvage by live cancer cells in the area as a DNA-seeking “antinuclear missile”. Antibody localization to extracellular nucleic acid waste helps mitigate concerns over target antigen depletion during therapy as tumor cell turnover and death yield a continuously renewing source of nucleic acids to draw ANADC to tumor microenvironments. Consistent with this, the greater efficacy of ANADC compared to the nonbinding ADC control in the MCF7 flank tumor model indicates nucleic acid targeting by DX3 enhances extracranial tumor bioavailability beyond what is conferred by nonspecific Fc interactions^8^ or the enhanced permeability and retention effect associated with solid tumors.^30^ Additionally, tumors sequestered behind the BBB are mostly invisible to other ADCs, but nucleoside salvage at the BBB facilitates ANA crossing into brain tumors.^18^ Here the biodistribution study confirming autoantibody uptake into brain tumors coupled with the survival benefit conferred by ANADC in the glioma model establish the potential for ANADC to be used against CNS malignancies. Overall, ANADC offers a tumor agnostic method for delivery of drugs to tumors that offers bioavailability to brain tumors and independence from requirements for specific surface or vascular targets or Fc-mediated uptake by resident tumor-associated macrophages.

Mechanistically, CatB is implicated in the intranuclear catabolism of DX3 by the findings that DX3 provokes CatB nuclear translocation and that the CatB inhibitor CA-074Me prolongs the intranuclear half-life of DX3. This contributed to the rationale for selecting a CatB-labile drug linker in developing ANADC, and the observed suppression of ANADC toxicity by CatB but not CatS inhibitors supports the conclusion that CatB is responsible for cleaving the Val-Cit linker in the nucleus of ANADC-treated cells. Based on this, the presence of functional CatB in cancer cells may be required for ANADC to mediate its effects on tumors. However, we cannot fully exclude the possibility that other proteases may also contribute to DX3 catabolism and/or ANADC Val-Cit linker cleavage. Although the CA-074Me inhibitor is active against CatB^24^ and DX3 does not cause notable translocation of the other cathepsins tested here, off-target inhibitor effects remain a possibility. In addition, the Val-Cit linker used here was originally developed to be specifically cleavable by CatB, but subsequent work indicates it may also be broken by a CatB-independent process.^26,27^ Additional studies are needed to elucidate specific mechanisms by which DX3 triggers the nuclear translocation by CatB, to determine if other proteases are involved in this process, and to elucidate the events following the initial nuclear penetration by ANADC that allow its apparent leakage into the cytoplasm at later time points.

In the present study there was no apparent increase in toxicity of ANADC compared to any controls, and DX3 tissue deposition at 96 h after treatment was not significantly increased in any of the examined tissues relative to the IgG control. We believe this is due to the mechanism of nucleoside salvage-mediated cellular penetration by DX3. The specific nucleoside transporter that facilitates membrane crossing by DX3, ENT2, is widely expressed in most normal cells and abundantly expressed in most malignancies. Normal cell expression of ENT2 raises concern over potential for off-target effects, but this is mitigated by the critical need for concurrent expression of ENT2 by cells and presence of extracellular DNA/nucleosides for salvage. This was previously established in work done with the prototype 3E10 antibody that evolved into DX3. In the absence of extracellular DNA/nucleosides, 3E10 cannot penetrate live cells. Consequently, we and others found that 3E10 preferentially localizes to areas enriched in extracellular DNA, such as necrotic tumors or areas of ischemic damage including stroke or myocardial infarction.^14,15,18,31,32^ This DNA-dependent mechanism of cellular penetration confers preferential antibody localization to tumor sites or damaged areas where live cells are using ENT2 to scavenge nucleosides from the abundant DNA/nucleoside substrates in the environment released by the dying cells and is observed to limit off-target tissue localization.

Overall, this work establishes an ANA-based strategy for delivery of reagents to cell nuclei that stems from ANA-induced CatB nuclear translocation. The MMAE cargo used in the present study was chosen for these proof-of-concept studies based on its known compatibility with CatB-labile linkers. We expect this approach will also facilitate intranuclear delivery of a wide range of cargoes, including other small molecule drugs, protein therapeutics, or nucleic acids for gene therapy.

## Methods

### Antibodies and Cell Lines

DX3 (PAT-DX3, Patrys Ltd., Melbourne, Australia) was designed as described in the results and generated in CHO cells and purified as previously described.^18^ IgG control was obtained from MedChemExpress (MCE, Monmouth Junction, NJ). U87 glioma, A549 lung cancer, HT29 colon cancer, and MDA-MB-231 and MCF7 breast cancer cells were obtained from ATCC (Manassas, VA, USA). DLD1 colorectal adenocarcinoma cells and primary NHAs were obtained from Horizon Discovery (Cambridge, UK) and Lonza (Morristown, NJ, USA), respectively.

### DNA Binding Assay

KD for DX3-DNA binding was determined by surface plasmon resonance using the Biacore T200 (Cytiva, Marlborough, MA, USA). The CM5 sensor chip in flow cells (FC) 1 and 2 was coated with neutravidin using the amine coupling kit (Cytiva, BR100050). FC1 was used as a reference cell. A 30-mer biotinylated single-stranded DNA ligand (0.025 μM, Sigma-Aldrich) was then immobilized onto the MC5 chip in FC2 through interaction with neutravidin. FC2 was used as the active flow cell. After three conditioning cycles with 5 M NaCl and six startup cycles with PBS pH 7.4, the analyte DX3 in PBS was added with a flow rate of 30 mL/min, contact time of 120 s, and dissociation time of 400 s. Results were analyzed using BIA Evaluation Software version 3.1 using a 1:1 fit kinetic binding analysis. The experiment was performed twice, with KDs of 113 nM and 112 nM determined in each experiment.

### Cell Penetration Assays

DLD1, U87, MCF7, A549, MDA-MB-231, or NHA cells were treated with 4 μM IgG control, ADC control, DX3, or ANADC for the indicated times, after which cells were washed, fixed in chilled 100% ethanol, and immunostained to detect antibody with Alexa Fluor 488 (AF488) conjugated secondary antibody detection and DAPI counterstain as previously described.^12,13,18^ Images were obtained using an EVOS fl digital fluorescence microscope (Advanced Microscopy Group, Bothell, WA, USA) and a Zeiss Axioimager m1microscope (Zeiss, Germany).

### Lysosomal, β-Tubulin, and Cathepsin Localization Assays

Cells treated with buffer control or 4 μM IgG control, DX3, or ANADC for the indicated times were washed, fixed, immunostained for LAMP1 (Invitrogen, 14-1079-80), β-tubulin (Invitrogen, 480011), CatB (Invitrogen, MA5-32651), CatK (Proteintech, 11239-1-AP), CatL (Invitrogen, BMS1032), or CatS (Invitrogen, PA5-82049), co-immunostained for IgG, subjected to DAPI nuclear counterstain, and visualized under fluorescence confocal microscopy. Nuclear overlap coefficients for cathepsin and DAPI nuclear channels were determined using ImageJ Colocalization Finder (NIH).

### Western Blots

Nuclear and cytoplasmic extracts of U87 glioma cells treated with 4 μM IgG control or DX3 were prepared using the NE-PER nuclear and cytoplasmic extraction reagents (Thermo Fisher Scientific) and probed for CatB using the CatB primary antibody described above, and signal detection was facilitated by HRP-conjugated secondary antibody. DX3 was probed by HRP-conjugated anti-human IgG Fc (abcam, Ab97225). Antibodies against Lamin B1 (Invitrogen, PA5-19468) or α-tubulin (Invitrogen, PA5-19489) were used for loading controls. Band intensities normalized to loading control were quantified by ImageJ.

### CatB and CatS Activity Assays

CatB and CatS enzymatic activity was measured using the cathepsin B inhibitor screening assay kit (BPS Bioscience, #79590) and cathepsin S inhibitor screening assay kit (BPS Bioscience, #79588), respectively, which use a fluorogenic cathepsin substrate to report enzymatic activity. Activity signal was quantified by a fluorescence plate reader (Synergy HT, BioTek) and expressed relative to reference purified CatB and CatS protein activity.

### Generation of ANADC and ADC Control

The drug monomethyl auristatin E was linked to nonbinding IgG isotype control or DX3 native cysteine thiol side chains using maleimidocaproyl (MC) joined to MMAE with a cleavable VC dipeptide linker and a *para*-aminobenzyloxycarbonyl (PABA) self-immolative spacer (MC-VC-PABA-MMAE). IgG or DX3 at 5 mg/mL was incubated with tris(2-carboxyethyl) phosphine (TCEP) at 37 °C for 2 h for reduction, followed by conjugation of MC-VC-PABA-MMAE to exposed thiols by incubation in 50 mM PBS pH 7.0 for 2 h at 4 °C. DAR, purity, and percentage of free drug were determined by HIC-HPLC, SEC-HPLC, and LC-MS. ADCs were >98% pure and had undetectable free drug. Minimal levels of endotoxin were confirmed by PTS cartridge.

### Protease Inhibitor Assays

For DX3 stability assays, U87 cells cultured in 12-well plates were treated with 4 μM DX3 in the presence of 10 μM CA-074Me CatB inhibitor (HY-100350, MCE) or an equivalent volume of DMSO for 1 h, after which media were removed and replaced with fresh media lacking DX3 but containing 10 μM CA-074Me CatB inhibitor or an equivalent volume of DMSO. Nuclear extracts were prepared from the cells at the time of media replacement (1 h after treatment with DX3) or 2 days after treatment and were probed for the presence of DX3 by western blot. Relative DX3 content was determined by ImageJ analysis of band intensities.

For morphology change assays, U87 cells cultured in 10 cm plates were treated with 10 μM protease inhibitors (E64 Cys protease inhibitor from the cathepsin B inhibitor screening assay kit, BPS Bioscience, #79590), CA-074Me CatB inhibitor (HY-100350, MCE), or LY3000328 CatS inhibitor (HY-15533, MCE) or media containing an equivalent volume of DMSO control for 3 h, followed by addition of control media or media containing 4 μM ANADC. Cell morphologies were monitored by bright-field microscopy for 60 min, and changes quantified by ImageJ.

### Clonogenic Assays

U87, MCF7, MDA-MB-231, DLD1, or A549 cells seeded at 1,000 cells per well in 6-well plates were treated with titrated doses of IgG control, ADC control, unconjugated DX3, or ANADC, and cell survival was evaluated by colony formation assay as previously described^11^ and plotted in Prism version 9.5.1.

### Biodistribution Study

Female NMRI-Foxn1^nu^ mice (Charles River) 6 weeks of age were subjected to stereotactic intracranial inoculation of 50,000 U87 glioma cells in 2 mL of PBS as previously described.^18^ Tumors were confirmed by volumetric MRI 2 weeks after inoculation. Mean volumes were 4.3 ± 0.1 mm^3^ at the time of the study. Mice were treated with tail vein injections of radiolabeled IgG control (*n* = 5) or DX3 (*n* = 5) corresponding to an activity of ∼16 MBq, and tissues were taken and analyzed for antibody uptake by gamma counter (Wizard 2, PerkinElmer, Finland) at 6 and 96 h (*n* = 2 and 3/group, respectively). Results are expressed as percentage of injected dose per gram of tissue analyzed (%ID/g).

### Tumor Studies

#### Triple-Negative MDA-MB-231 Breast Cancer Xenograft Study

Female NOG mice were injected subcutaneously with 5 × 10^6^ MDA-MB-231 cells in Matrigel to generate flank tumors. Once tumors reached a mean volume of ∼250 mm^3^, mice were randomized to groups for IP treatment with vehicle control (*n* = 8) or ANADC at 1, 5, or 10 mg/kg (*n* = 8/group) once a week for 4 weeks. Tumor volumes were tracked by caliper measurements, and mouse weights and behaviors monitored throughout the study.

#### ER+ MCF7 Breast Cancer Xenograft Study

Female Balb/c nude mice were administered 60-day release 17-β-estradiol pellets and 3 days later injected subcutaneously with 5 × 10^6^ MCF7 cells in Matrigel to generate flank tumors. Once tumors reached a mean volume of ∼150 mm^3^, mice were randomized to groups for IP treatment with vehicle control (*n* = 6), ADC control (10 mg/kg) (*n* = 6), unconjugated DX3 (10 mg/kg) (*n* = 6), or ANADC (10 mg/kg) (*n* = 6) once a week for 4 weeks. Tumor volumes were tracked by caliper measurements, and mouse weights and behaviors monitored throughout the study.

#### HT29 Colon Cancer Xenograft Study

Female Balb/c nude mice were injected subcutaneously with 5 × 10^6^ HT29 cells in Matrigel to generate flank tumors. Once tumors reached a mean volume of ∼150 mm^3^, mice were randomized to groups for IP treatment with vehicle control (*n* = 6), unconjugated DX3 (10 mg/kg) (*n* = 6), or ANADC (10 mg/kg) (*n* = 6) once a week for 4 weeks. Tumor volumes were tracked by caliper measurements, and mouse weights and behaviors monitored throughout the study.

#### U87 Orthotopic Glioma Survival Study

Orthotopic U87 glioma tumors were established in female athymic nude mice 6 weeks of age by intracranial inoculation of 5 × 10^4^ luciferase-expressing cells as previously described.^18^ One week after inoculation mice were evaluated for tumors by IVIS and then randomized into groups for four weekly treatments by tail vein injection of control buffer (*n* = 6), unconjugated DX3 (*n* = 5), and ANADC (*n* = 5). Mice were closely observed and euthanized for humane end points of neurologic or behavior changes or weight loss. Kaplan–Meier survival curves were plotted in Prism 9.5.1.

All mouse studies were conducted under an IACUC-approved protocol, and mice were humanely euthanized for end points of tumor size, weight loss, or behavior changes.

### Statistical Analysis

*P* values were determined as indicated by Student’s *t* test, Tukey’s multiple comparisons test, or log-rank test in Prism 9.5.1. *P* < 0.05 was considered significant.

### Safety

No unexpected or unusually high safety hazards were encountered in this work.

## Supporting Information Available

Figure S1. Cathepsin localization in cells treated with control buffer, IgG control, or DX3. Figure S2. DX3 induces CatB translocation from the cytoplasm. Figure S3. DX3 induces nuclear accumulation of CatB in a panel of cancer cells. Figure S4. DX3 induces nuclear accumulation in primary NHAs. Figure S5. Determination of ANADC DAR. Figure S6. ANADC penetrates cells and progressively impacts morphologies. Figure S7. ANADC localizes exclusively in nuclei without β-tubulin overlap at early time points. Figure S8. ANADC localizes exclusively in nuclei without lysosomal overlap at early time points. Figure S9. ANADC cytotoxicity is suppressed by CatB inhibition. Figure S10. ANADC penetrates and is toxic to a panel of cancer cells. Figure S11. Comparison of IgG control and DX3 distribution in normal brain outside of tumor (PDF)
