# Supplementary material for: Cathepsin B Nuclear Flux in a DNA-Guided “Antinuclear Missile” Cancer Therapy
Source: ACS Cent Sci. 2024 Jul 15;10(8):1562–72. doi: 10.1021/acscentsci.4c00559 (PMC11363321; doi:10.1021/acscentsci.4c00559)
Supplement: Supplementary file 1 — oc4c00559_si_001.pdf [file oc4c00559_si_001.pdf]

Supporting Information for:

Cathepsin B nuclear flux in a DNA-guided "antinuclear missile" cancer therapy

Fei Cao[1], Caroline Tang[1], Xiaoyong Chen[1], Zewei Tu[2], Ying Jin[3], Olivia M. Turk[1], Robert N. Nishimura[4,5], Allen Ebens[6], Valentina Dubljevic[7], James A. Campbell[7], Jiangbing Zhou[2,8], James E. Hansen[1,8*]

[1]Department of Therapeutic Radiology, Yale School of Medicine; New Haven, CT, USA
[2]Department of Neurosurgery, Yale School of Medicine; New Haven, CT, USA
[3]Division of Vascular Surgery and Endovascular Therapy; Department of Surgery, Yale School of Medicine; New Haven, CT, USA
[4]Department of Research & Development, Greater Los Angeles Veterans Affairs Healthcare System; Los Angeles, CA, USA
[5]Department of Neurology, David Geffen School of Medicine at UCLA; Los Angeles, CA
[6]Adanate; Palo Alto, CA, USA
[7]Patrys Ltd; Melbourne, Australia
[8]Yale Cancer Center; New Haven, CT, USA

*Corresponding author. Email: james.e.hansen@yale.edu

Pages: 17

Figures: 11 (Figures S1-S11)

Tables: N/A

Figure S1

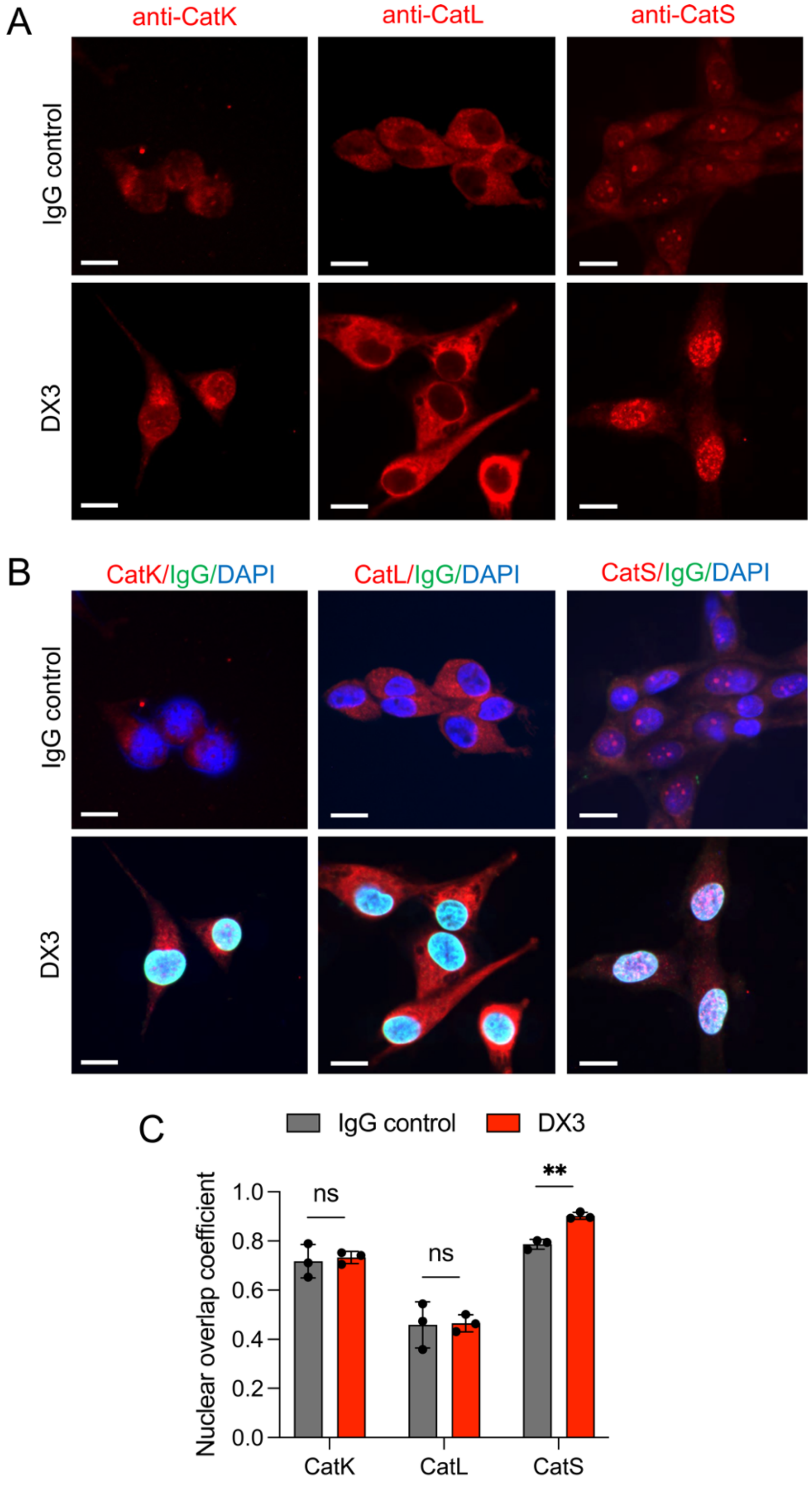
A
anti-CatK
anti-CatL
anti-CatS
IgG control
DX3
B
CatK/IgG/DAPI
CatL/IgG/DAPI
CatS/IgG/DAPI
IgG control
DX3
C
IgG control
DX3
Nuclear overlap coefficient
1.0
0.8
0.6
0.4
0.2
0.0
ns
ns
**
CatK
CatL
CatS

Figure S1. Cathepsin localization in cells treated with control buffer, IgG control, or DX3. (A, B) U87 glioma cells treated for 15 minutes with control buffer or 4 μM IgG control or DX3 were immunostained for CatB, K, L, or S (red), IgG (green), with blue DAPI nuclear counterstain. Representative confocal immunofluorescence microscopy images are shown, including cathepsin single channel (**A**) and merged images of DAPI, anti-IgG, and anti-cathepsin (**B**). Bars: 10 μm. (**C**) Cathepsin nuclear overlap coefficients. Nuclear overlap coefficients for CatK, L, and S in U87 glioma cells treated with IgG control or DX3 were determined by ImageJ Colocalization Finder. DX3 did not cause any significant changes compared to IgG control in nuclear signal for CatK or L and had only a small effect on CatS. **P<0.01, Tukey's multiple comparisons test, n=3.

Figure S2

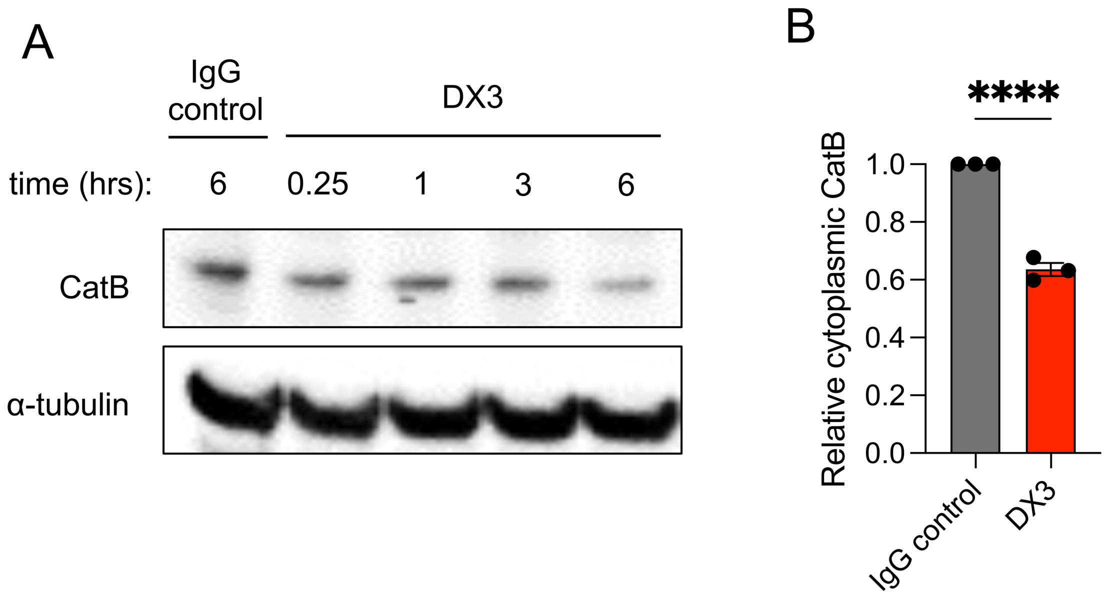

Figure S2. DX3 induces CatB translocation from the cytoplasm. (A, B) Cytoplasmic contents isolated from U87 glioma cells treated for 0-6 hours with 4 μM IgG control or DX3 were analyzed by western blot probed for CatB, with tubulin for loading control. As shown in the main manuscript figures, DX3 caused a significant increase in CatB nuclear content, and here a corresponding decrease in cytoplasmic content of the active form of CatB (~29 kDa) is shown. Representative western blot on nuclear extracts is shown in (**A**), and ImageJ quantification of CatB cytoplasmic content relative to content in cells treated with IgG control at 6 hours is shown in (**B**). ****$P<0.0001$, two-tailed student's t-test, n=3.

Figure S3

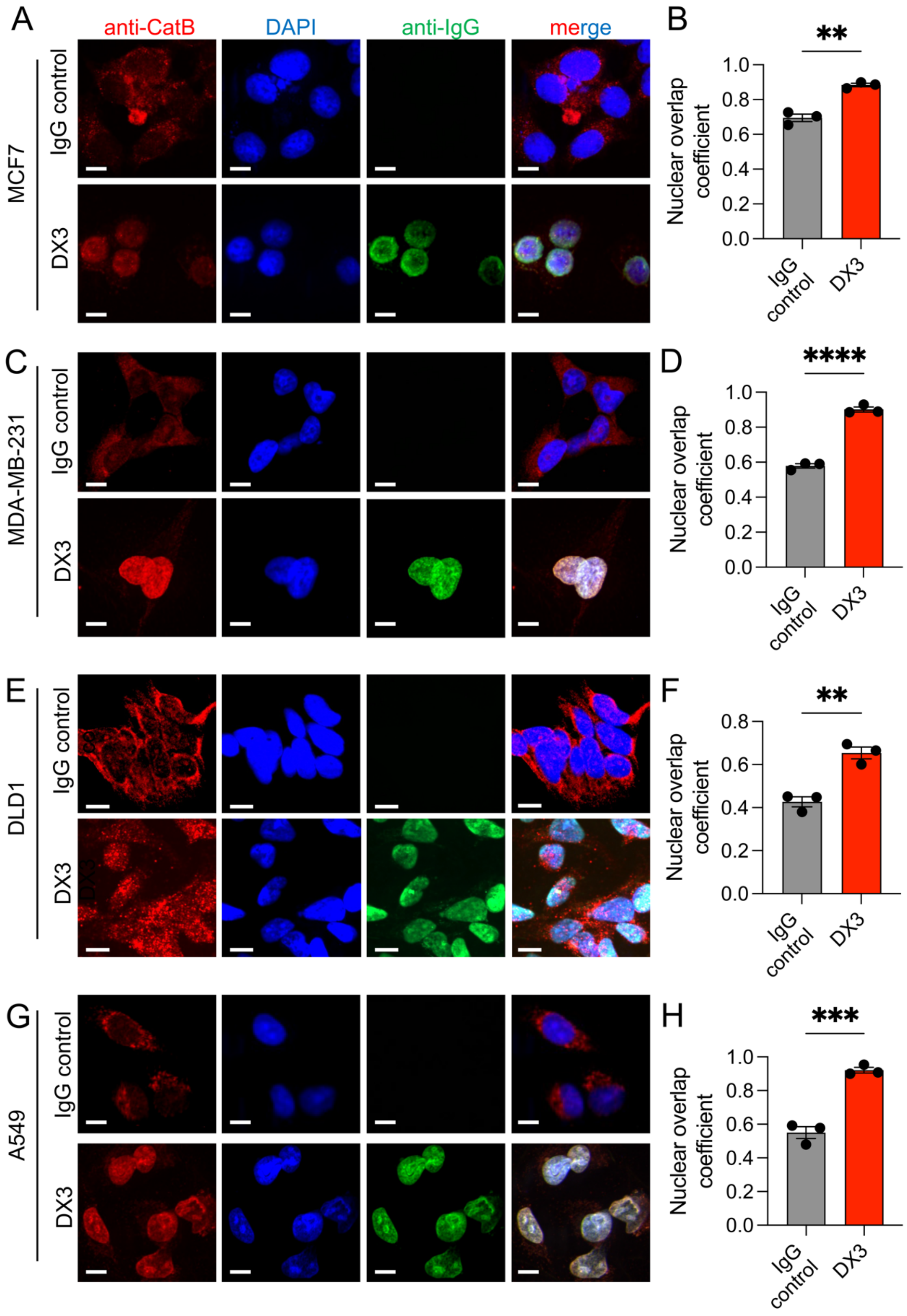

A
anti-CatB
DAPI
anti-IgG
merge
MCF7
IgG control
DX3
B
**
Nuclear overlap coefficient
1.0
0.8
0.6
0.4
0.2
0.0
IgG control
DX3
C
MDA-MB-231
D
****
E
DLD1
F
G
A549
H
***

Figure S3. DX3 induces nuclear accumulation of CatB in a panel of cancer cells. (A-H) Representative immunofluorescence confocal microscopy images of CatB (red), DAPI (blue), and anti-IgG (green) in a panel of cancer cells treated with 4 μM IgG control or DX3 for 24 hours are shown, along with corresponding nuclear overlap coefficients for CatB and DAPI signals determined by ImageJ. (**A, B**) ER+ MCF7 breast cancer cells. (**C, D**) Triple negative MDA-MB-231 breast cancer cells. (**E, F**) DLD1 colon cancer cells. (**G, H**) A549 lung adenocarcinoma cells. Bars: 20 μm. **P<0.01, ***P<0.001, ***P<0.0001, two-tailed student's test, n=3.

Figure S4

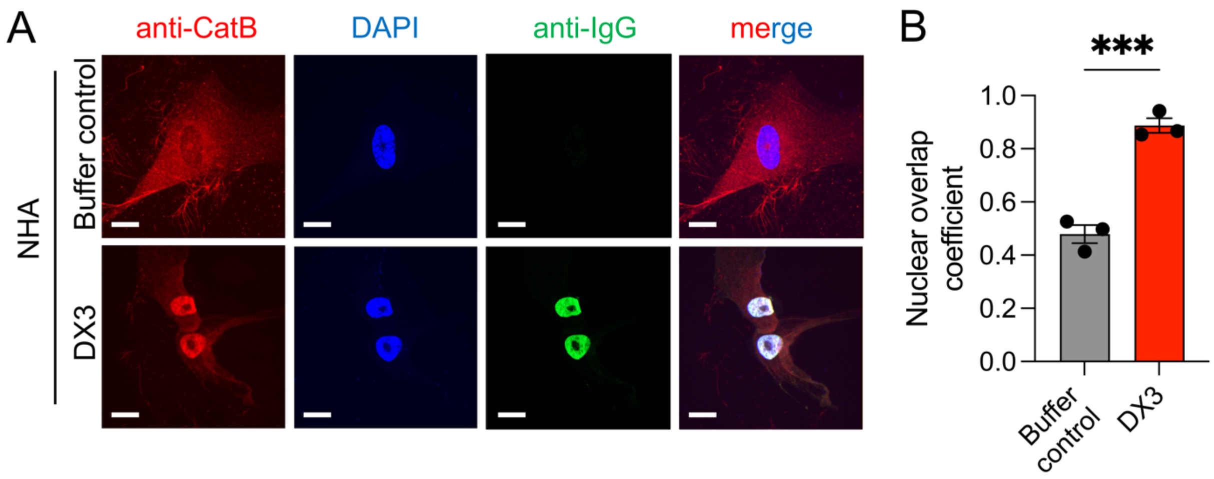

Figure S4. DX3 induces nuclear accumulation in primary NHAs. (A, B) Representative immunofluorescence confocal microscopy images of NHAs treated with control buffer or DX3 immunostained for CatB (red), DAPI (blue), IgG (green), and merged images are shown in (**A**), and nuclear overlap coefficients measured by ImageJ Colocalization Finder to determine overlap coefficient for CatB and DAPI channels in (**B**). Bars: 10 μm. ***$P<0.001$, two-tailed student's test, n=3.

Figure S5

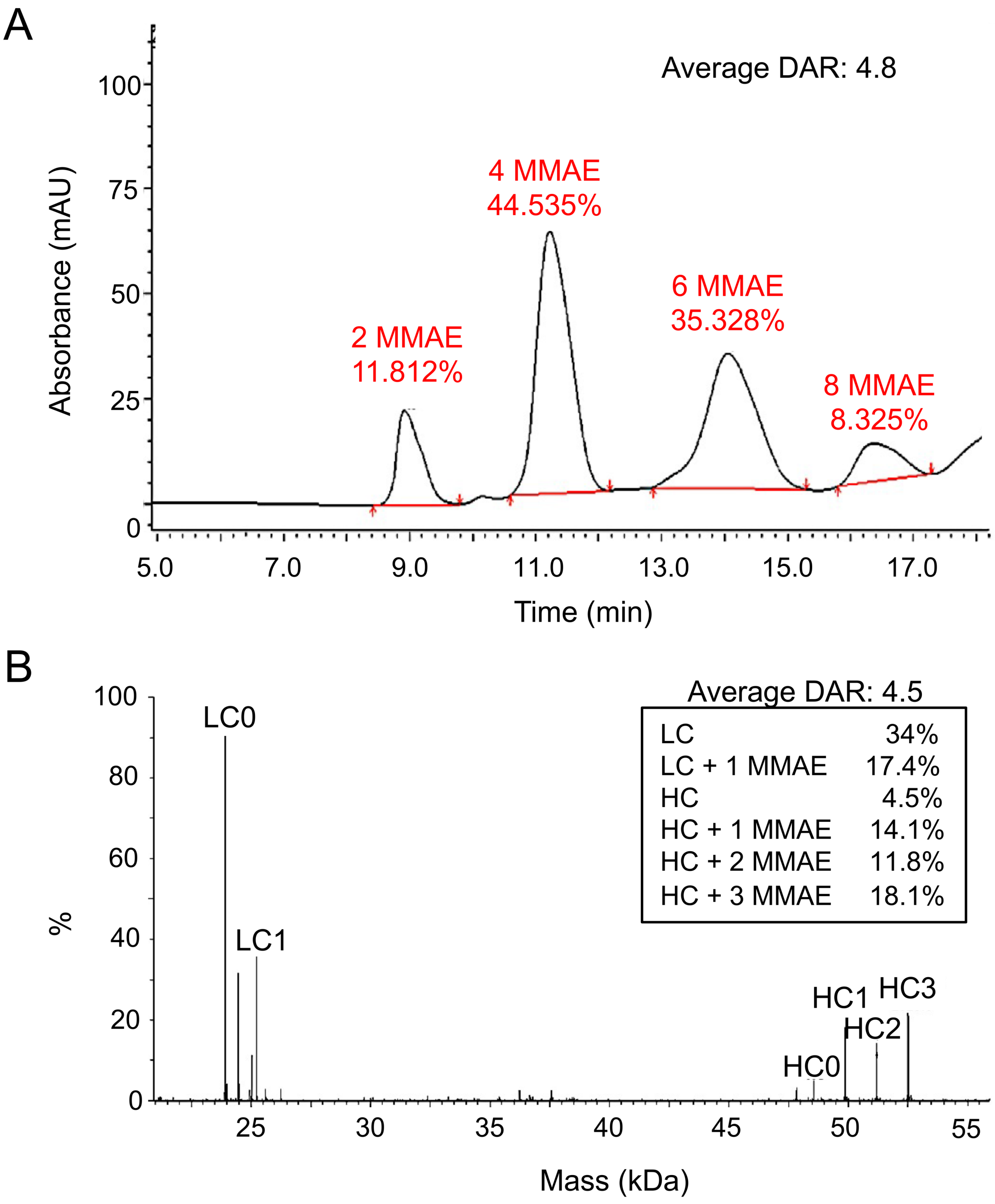

Figure S5. Determination of ANADC DAR. (A) ANADC HIC profile shows mean DAR 4.8, with four linked MMAE molecules the predominant species. (**B**) ANADC LC-MS profile shows mean DAR 4.5, consistent with the HIC results.

Figure S6

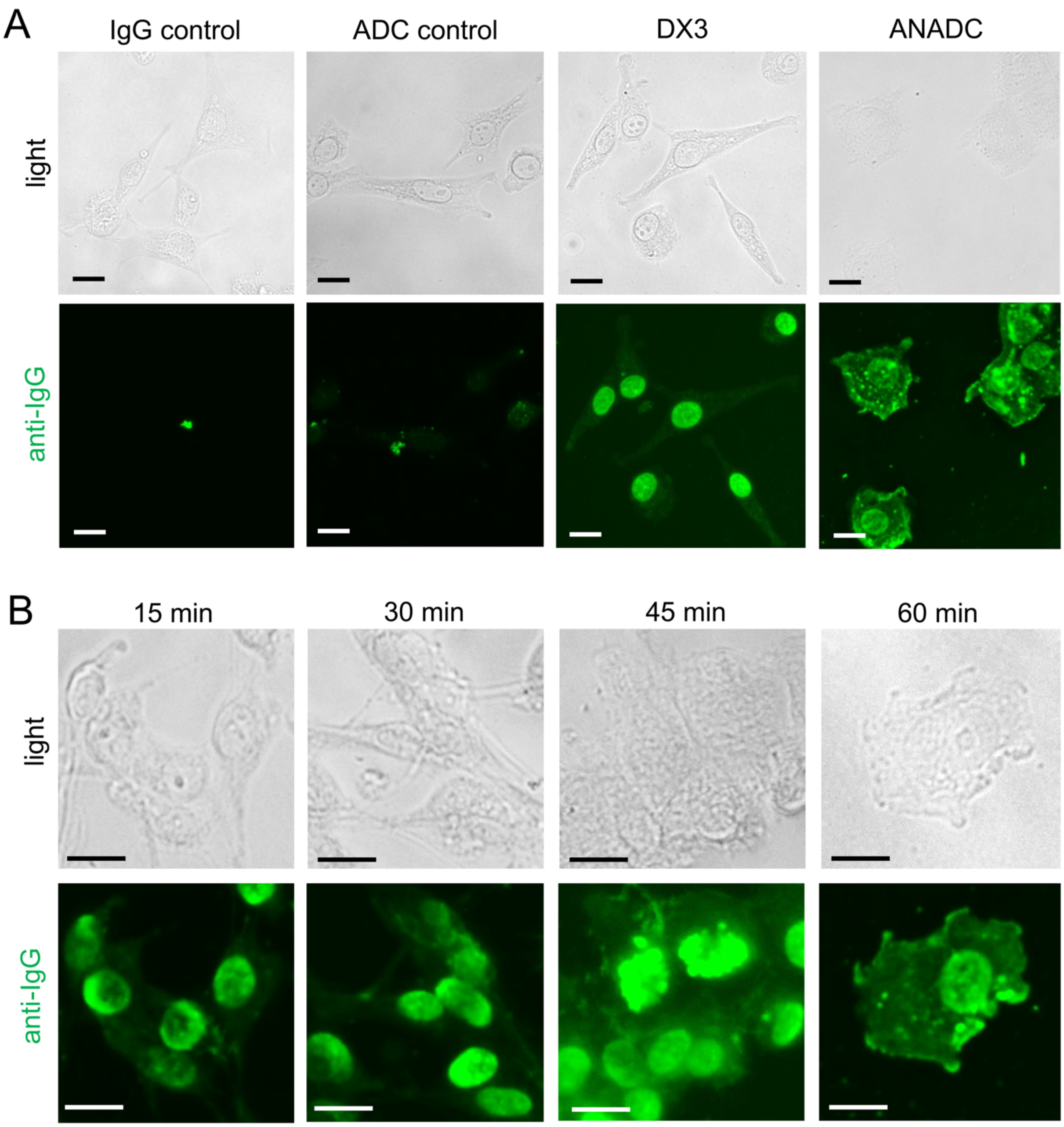

Figure S6. ANADC penetrates cells and progressively impacts morphologies. (A) U87 cells treated with 4 μM IgG control, ADC control, unconjugated DX3, or ANADC were visualized by bright-field and anti-IgG immunofluorescence (green) microscopy. Representative images at 60 minutes

after treatment show minimal to no uptake of IgG control and ADC control, while DX3 and ANADC showed significant penetration into cells. Compared to DX3, cells treated with ANADC showed morphologic changes and an IgG staining pattern consistent with DX3 escape from the nucleus into the cytoplasm, suggestive of dissolution of the nuclear membrane caused by cell death. Bars: 20 μm. (**B**) U87 cells treated with 4 μM ANADC imaged by bright-field and anti-IgG fluorescence microscopy at the indicated timepoints after treatment show progressive loss of cell architecture and subsequent escape of DX3 signal from the nucleus into the cytoplasm, consistent with MMAE-induced toxicity. Bars: 20 μm.

Figure S7

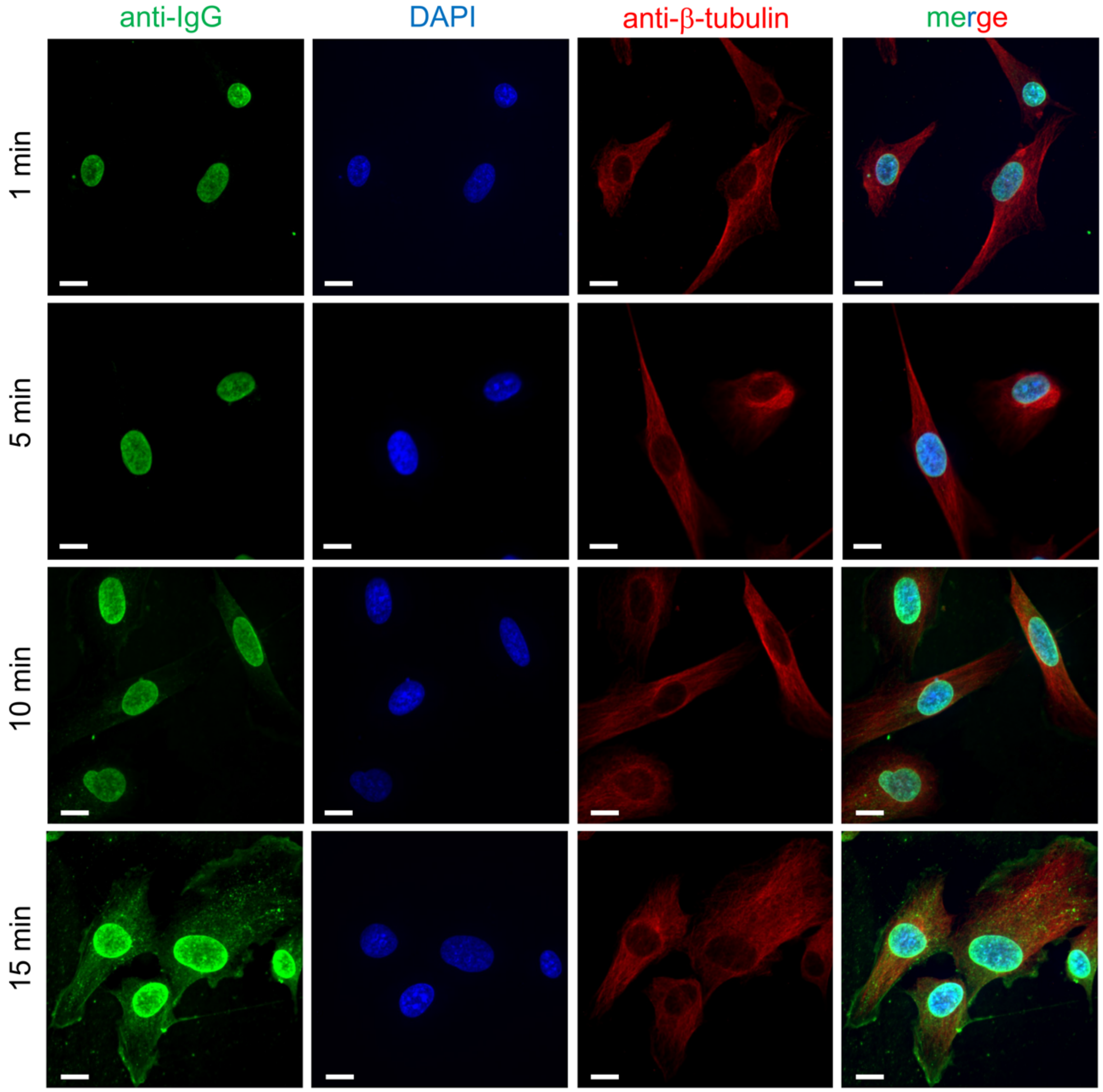

Figure S7. ANADC localizes exclusively in nuclei without β-tubulin overlap at early time points. U87 cells treated with 4 μM ANADC were visualized by anti-IgG (green), DAPI (blue), and anti-β-tubulin (red) confocal fluorescence microscopy at 1, 5, 10, and 15 minutes after ANADC exposure to the cells. Representative single channel and merged images show at early time points (1 and 5 min) ANADC is exclusively localized in the nucleus and does not overlap

with β-tubulin. Cytoplasmic appearance of ANADC signal progressively occurs after 10 min. Bars: 10 μm.

Figure S8

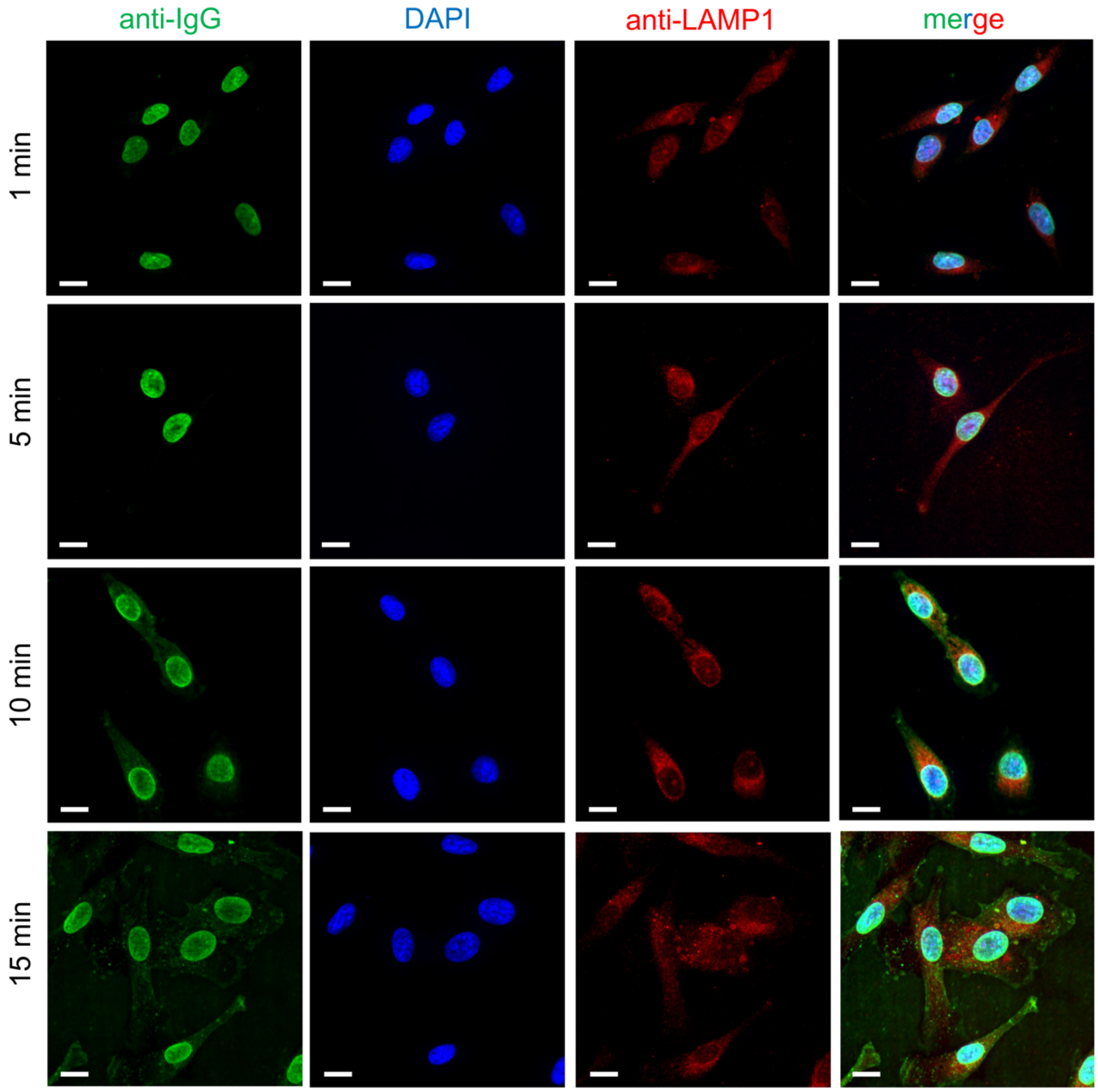

Figure S8. ANADC localizes exclusively in nuclei without lysosomal overlap at early time points. U87 cells treated with 4 μM ANADC were visualized by anti-IgG (green), DAPI (blue), and anti-LAMP1 (red) confocal fluorescence microscopy at 1, 5, 10, and 15 minutes after ANADC exposure to the cells. Consistent with **Fig. S6 and S7**, representative single channel and merged images show at early time points (1 and 5 min) ANADC is exclusively localized in the nucleus and does

not overlap with LAMP1. As previously noted, cytoplasmic appearance progressively occurs after 10 min. Bars: 10 μm.

Figure S9

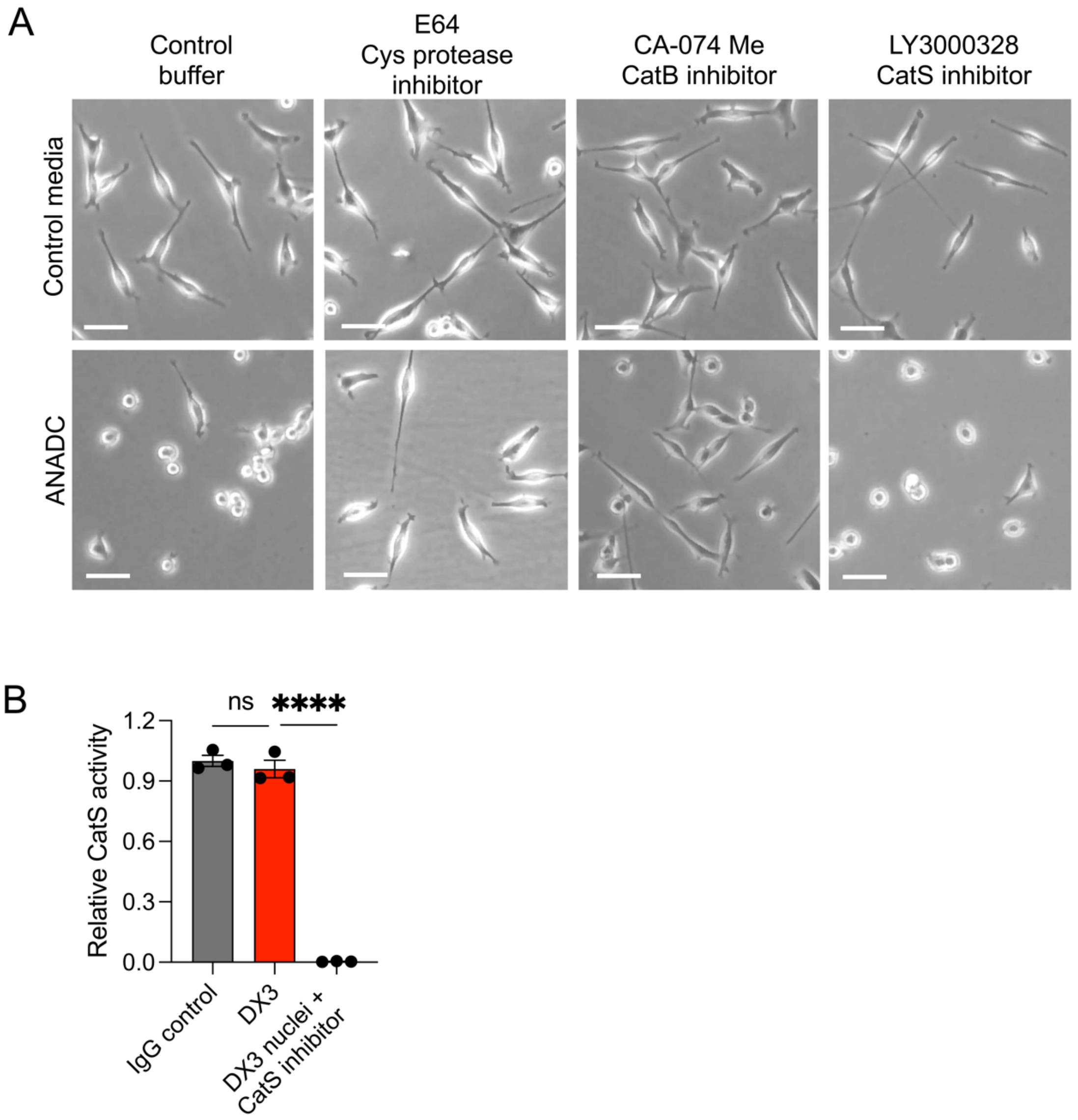

Figure S9. ANADC cytotoxicity is suppressed by CatB inhibition. (**A**) Representative bright-field microscopy images of U87 glioma cells 45 minutes after treatment with control media or 4 μM ANADC in the presence of control buffer, E64 Cys protease inhibitor, CA-074Me CatB inhibitor, or LY3000328 CatS inhibitor are shown. Cell morphologies were rapidly lost after ANADC treatment in control buffer or with CatS inhibitor but were maintained in the presence of the broad spectrum

Cys protease inhibitor or the CatB inhibitor, consistent with CatB-dependent cytotoxicity. Bars: 40 μm. (**B**) Confirmation of CatS inhibition. Nuclear extracts from cells treated with IgG control or DX3 ± CatS inhibitor as described in (**A**) were tested for CatS activity by assaying the nuclear extracts for cleavage of a CatS fluorogenic substrate measured by fluorescence plate reader. ****P<0.0001, two-tailed student's t-test, n=3.

Figure S10

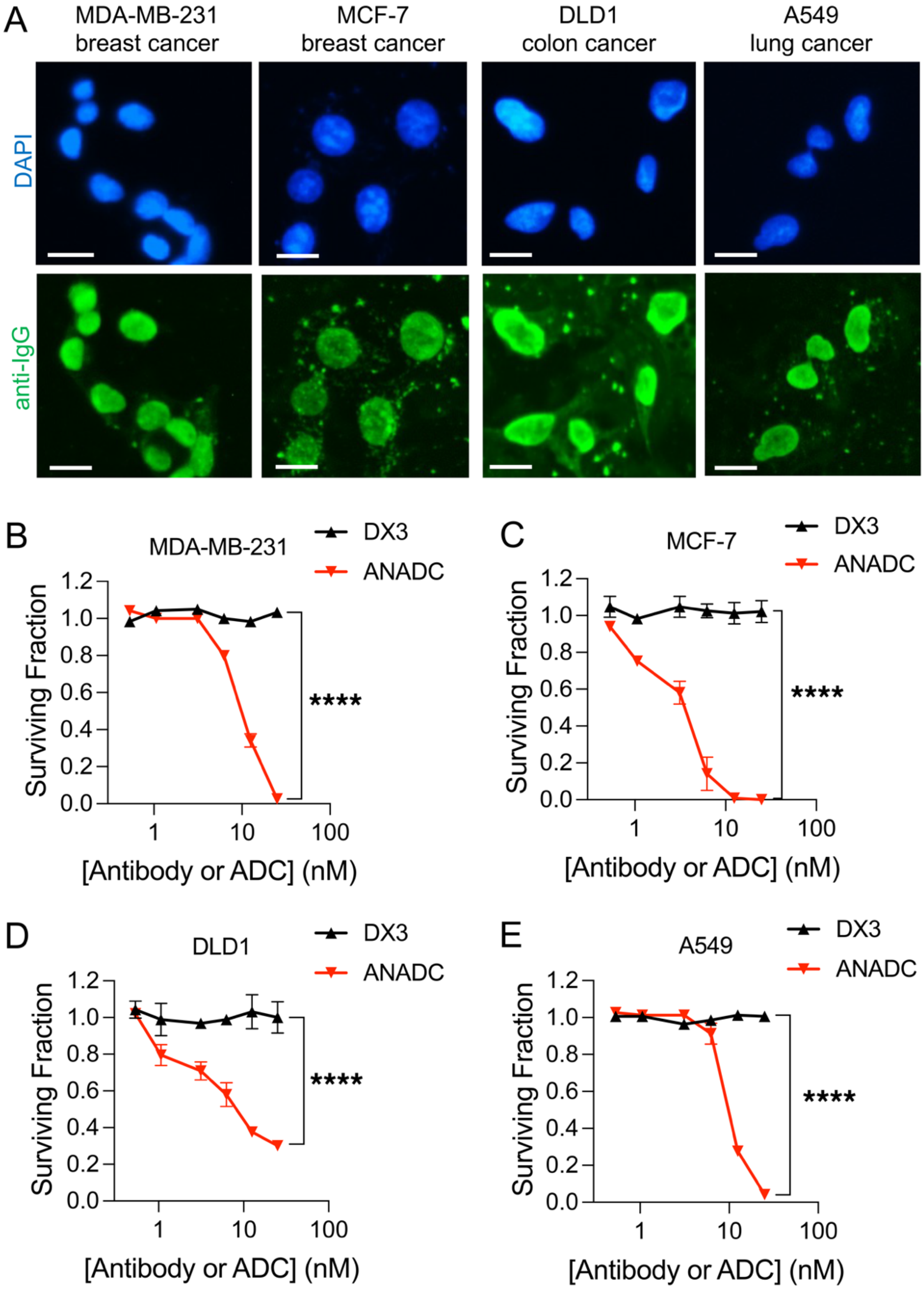
A
MDA-MB-231
breast cancer
MCF-7
breast cancer
DLD1
colon cancer
A549
lung cancer
DAPI
anti-IgG
B
MDA-MB-231
DX3
ANADC
Surviving Fraction
[Antibody or ADC] (nM)
****
C
MCF-7
DX3
ANADC
Surviving Fraction
[Antibody or ADC] (nM)
****
D
DLD1
DX3
ANADC
Surviving Fraction
[Antibody or ADC] (nM)
****
E
A549
DX3
ANADC
Surviving Fraction
[Antibody or ADC] (nM)
****

Figure S10. ANADC penetrates and is toxic to a panel of cancer cells. (A) Representative immunofluorescence confocal microscopy images of DAPI (blue) and anti-IgG (green) in a panel of cancer cells (triple-negative MDA-MB-231 and ER+ MCF7 breast cancer, DLD1 colon cancer, and A549 lung adenocarcinoma) treated with 4 μM ANADC are shown. Bars: 10 μm. (**B**) Cells were treated with titrated doses of DX3 or ANADC and surviving fractions determined by colony formation assay. ****P<0.0001, two-tailed student's t-test, n=3.

Figure S11

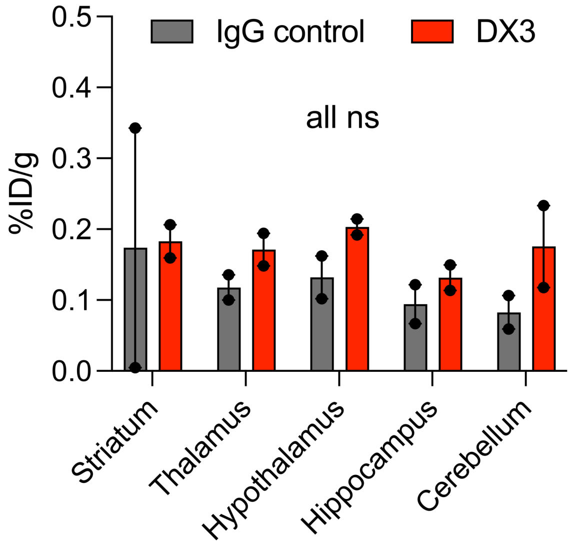

Figure S11. Comparison of IgG control and DX3 distribution in normal brain outside of tumor. Brains were taken from mice bearing intracranial U87 glioma tumors six hours after treatment with radiolabeled IgG control or DX3. Antibody uptake in specific regions of brain outside of the tumor was compared. No significant difference in distribution was detected. Results are expressed as %ID/g. Tukey's multiple comparisons test, n=2.
